# Biophysical Properties of Escherichia coli Cytoplasm in Stationary Phase by Superresolution Fluorescence Microscopy

**DOI:** 10.1128/mBio.00143-20

**Published:** 2020-06-16

**Authors:** Yanyu Zhu, Mainak Mustafi, James C. Weisshaar

**Affiliations:** a Department of Chemistry, University of Wisconsin−Madison, Madison, Wisconsin, USA; Massachusetts Institute of Technology

**Keywords:** *E. coli* stationary phase, diffusive properties, nucleoid morphology, spatial distributions, superresolution fluorescence microscopy

## Abstract

In nature, bacteria must survive long periods of nutrient deprivation while maintaining the ability to recover and grow when conditions improve. This quiescent state is called stationary phase. The biochemistry of Escherichia coli in stationary phase is reasonably well understood. Much less is known about the biophysical state of the cytoplasm. Earlier studies of harvested nucleoids concluded that the stationary-phase nucleoid is “compacted” or “supercompacted,” and there are suggestions that the cytoplasm is “glass-like.” Nevertheless, stationary-phase bacteria support active transcription and translation. Here, we present results of a quantitative superresolution fluorescence study comparing the spatial distributions and diffusive properties of key components of the transcription-translation machinery in intact E. coli cells that were either maintained in 2-day stationary phase or undergoing moderately fast exponential growth. Stationary-phase cells are shorter and exhibit strong heterogeneity in cell length, nucleoid volume, and biopolymer diffusive properties. As in exponential growth, the nucleoid and ribosomes are strongly segregated. The chromosomal DNA is locally more rigid in stationary phase. The population-weighted average of diffusion coefficients estimated from mean-square displacement plots is 2-fold higher in stationary phase for both RNA polymerase (RNAP) and ribosomal species. The average DNA density is roughly twice as high as that in cells undergoing slow exponential growth. The data indicate that the stationary-phase nucleoid is permeable to RNAP and suggest that it is permeable to ribosomal subunits. There appears to be no need to postulate migration of actively transcribed genes to the nucleoid periphery.

## Abstract (precis)

Bacteria in nature usually lack sufficient nutrients to enable growth and replication. Such starved bacteria adapt into a quiescent state known as the stationary phase. The chromosomal DNA is protected against oxidative damage, and ribosomes are stored in a dimeric structure impervious to digestion. Stationary-phase bacteria can recover and grow quickly when better nutrient conditions arise. The biochemistry of stationary-phase E. coli is reasonably well understood. Here, we present results from a study of the biophysical state of starved E. coli. Superresolution fluorescence microscopy enables high-resolution location and tracking of a DNA locus and of single copies of RNA polymerase (the transcription machine) and ribosomes (the translation machine) in intact E. coli cells maintained in stationary phase. Evidently, the chromosomal DNA remains sufficiently permeable to enable transcription and translation to occur. This description contrasts with the usual picture of a rigid stationary-phase cytoplasm with highly condensed DNA.

## INTRODUCTION

Bacteria in nature spend the vast majority of their time in a quiescent state induced by lack of nutrients. In response to starvation, Gram-negative bacteria such as Escherichia coli enter stationary phase, a state of low metabolic activity that protects cells from starvation and other stresses for many days (1). In stationary phase, cells cease to divide but maintain the potential to recover when nutrient levels subsequently improve.

Much has been learned about the biochemistry of stationary-phase bacteria, especially E. coli. Upregulation of the alternative RNA polymerase (RNAP) subunit σ^S^ and of certain transcriptional regulators leads to dramatic changes in transcription and translation profiles as well as in cell morphology, membrane composition, and metabolic state (1–5). The overall translation rate slows dramatically. Protease production increases, enabling the cell to use existing proteins as a source of amino acids (2). Many of the 70S ribosomes that survive degradation are stored as inert 100S ribosome dimers driven by the expression of the proteins ribosome modulation factor (RMF) and hibernation promoting factor (HPF) (2, 6–8). These 100S dimers can quickly revert to functional 70S monomers and 30S and 50S subunits when external nutrient conditions improve, enabling a rapid transition back to normal growth (7, 9). A third hibernation factor, ribosome-associated inhibitor A (RaiA), can stabilize the 70S ribosome in an inactive state (2, 7).

In early- and late-stationary-phase E. coli, the DNA-binding protein Dps becomes highly abundant; the copy number per cell may rise to 100,000 or more (10, 11). The general view of the nucleoid in stationary phase holds that ring-shaped, dodecameric Dps oligomers combine with Mg^2+^ and the chromosomal DNA to form microcrystals or nanocrystals believed to protect the DNA from oxidative stress (12, 13). Researchers have described the stationary-phase nucleoid as “compacted” (1), “supercondensed” (14), or “tightly compacted” (15, 16). It has been suggested that the cytoplasm adopts “glass-like” properties (17). However, this picture of the nucleoid is typically based on a 2004 report of a study that used atomic force microscopy (AFM) and fluorescence microscopy to characterize nucleoids that had been extracted from cells and dried on glass (16). For nucleoids harvested from exponential-phase cells, the images showed narrow 40 nm and 80 nm fibers emanating from concentrated blobs (see Fig. 2 in reference 16). For nucleoids harvested from early and late stationary phase, the nucleoid images became more and more compact (16). From studies in live cells and *in vitro*, we know that the overall size of the confined DNA polymer is sensitive to transcription and translation activity, salt concentration, confinement, crowding, the nature and concentration of DNA-binding proteins, and the presence of polycationic species (14, 18–21). In our view, harvested and dried nucleoids are unlikely to report reliably on the natural state of the nucleoid in intact cells. Instead, they may primarily reflect how readily the real nucleoid structure is disrupted by treatment with lysozyme, detergent, distilled water, drying, etc.

Two recent studies obtained fluorescence images of the nucleoid in intact cells after starvation. Sanyal and coworkers (22) performed widefield fluorescence studies of DNA and of ribosomes in intact stationary-phase cells. They observed strong DNA-ribosome segregation and predominantly single-lobed nucleoids of widely differing lengths. Meyer and coworkers (23) compared nucleoid images between wild-type (WT) and Δ*dps* cells in 24-h and 96-h stationary phase. The ratio of nucleoid length to overall cell length was about 20% lower in the WT cells that expressed Dps normally.

In our view, the most useful measure of the overall degree of DNA compaction in an intact cell is the mean density of DNA, which we express in units of base pairs per cubic micrometer. This quantity provides a key input for theoretical efforts to model diffusion within the E. coli nucleoid (24). We know of no previous work that quantitatively compared nucleoid volume and DNA density in the stationary phase versus the exponential-growth phase. Kuhlman and Cox measured the average curvature of DNA images obtained by DAPI staining in stationary phase (25), but their results do not provide estimates of absolute DNA densities.

In this work, we used superresolution, single-molecule fluorescence microscopy (26–28) to quantitatively compare the biophysical properties of key components of the E. coli transcriptional and translational machinery in 2-day stationary phase and in moderately fast exponential growth (VH1000 strain; doubling time, 45 ± 1 min at 30°C). Two-day stationary-phase cells that had been incubated for 48 h in EZ rich defined medium (EZRDM) were plated on a glass coverslip in a microfluidic chamber and maintained in stationary phase by the use of a steady flow of “spent medium,” the supernatant fluid harvested from a separate 2-day stationary-phase culture (29). Spent medium prevents any noticeable cell growth during observation under the microscope for periods of at least 1 to 2 h. The same cells remain viable; they begin to grow within ∼1 min of introduction of a flow of fresh growth medium (see Fig. S1 in the supplemental material). Short cells and long cells begin to grow on comparable timescales.

10.1128/mBio.00143-20.2 FIG S1 Recovery of three single stationary-phase cells after restoration of growth medium. Stationary-phase cells were plated in the microfluidic device. Spent medium flowed for the first 30 min. At *t *= 0, the flow was switched to fresh, aerated EZRDM. Phase-contrast images were acquired every 12 s to measure cell length. *L*/*L*_0_ is the cell length versus time, normalized to its length at *t* = –30 min (i.e., long before the flow was changed). There was no evidence of growth seen while flowing the spent medium. Download FIG S1, EPS file, 1.2 MB. Copyright © 2020 Zhu et al. 2020 Zhu et al. This content is distributed under the terms of the Creative Commons Attribution 4.0 International license.

We obtained projected two-dimensional (2D) superresolution spatial distributions of DNA (labeled by HU-PAmCherry), RNAP (β′ subunit labeled by mEos2), ribosomal species (labeled by L9-mEos2), and the exogenous fluorescent protein Kaede tetramer (expressed from a plasmid). Single copies were located to an accuracy of σ = 32 to 105 nm, depending on the biomolecule under study. In addition, we analyzed diffusive or subdiffusive motion from single-particle trajectories of RNAP, ribosomes, and Kaede as well as the DNA locus *Right2* (labeled using the ParB-*ParS* scheme).

In contrast to the usual picture of the stationary-phase nucleoid as a supercompacted, relatively impermeable structure, we found that RNAP (450 kDa), Kaede tetramer (110 kDa), and the more rapidly diffusing ribosomal species (probably including 50S subunits, 1,500 kDa) readily permeated the 2-day stationary-phase nucleoid. This is consistent with the need for a small but significant level of transcription and translation activity in stationary phase (23). RNAP is largely sequestered within the nucleoid in both stationary phase and exponential growth.

Surprisingly, the population-weighted average of RNAP diffusion coefficients was twice as high in stationary-phase cells as in exponentially growing cells. On average, on a 10-s timescale, the local jiggling motion of the DNA locus *Right2* was found to be attenuated 4-fold in stationary phase compared with exponential growth. The DNA polymer is evidently less flexible in stationary phase. The population-weighted average diffusion coefficient of ribosomal species on a 180-ms timescale was found to be twice as large in stationary phase as in exponential growth. In exponential growth phase, the more slowly diffusing ribosomal species tend to be located outside the nucleoid, while the more rapidly diffusing ribosomal species tend to locate inside. This behavior has been interpreted to mean that large 70S polysomes (the predominant species) are excluded from the nucleoid, while the smaller 30S and 50S subunits readily penetrate the nucleoid (30). This enables initiation and elongation of cotranscriptional translation throughout the nucleoid. The same distribution patterns of fast and slow ribosomal components also occur in stationary phase. However, the assignments are less obvious because a wide variety of ribosomal species may be present.

Finally, we provide quantitative estimates of the volume of the stationary-phase nucleoid and the density of its chromosomal DNA (expressed in base pairs per cubic micrometer) and compare these quantities with estimates of volume and DNA density from our earlier study of cells growing slowly in minimal medium (147 min doubling time) (31). On average, the DNA density was found to be roughly 2-fold higher in the stationary-phase cells than in the slowly growing cells. For E. coli, the new data suggest that the nucleoid is indeed somewhat denser in 2-day stationary phase than in exponential growth but remains sufficiently permeable and flexible to enable transcription to occur throughout the nucleoid volume.

## RESULTS

### Morphology of 2-day stationary-phase E. coli.

**FIG 1:**
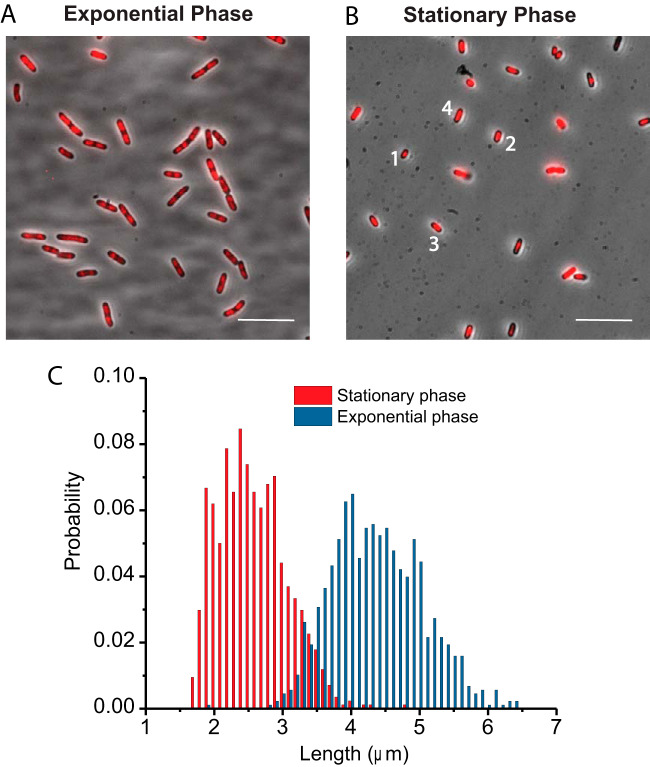
(A and B) Fluorescence images of E. coli nucleoid stained with Sytox Green superimposed on phase-contrast images (A) for cells in exponential phase and (B) for cells in 48-h stationary phase. Scale bars: 10 μm. The cells labeled 1 through 4 in panel B have tip-to-tip lengths estimated from Oufti of 1.94 μm, 2.32 μm, 2.33 μm, and 2.83 μm, respectively. (C) Length distribution of WT VH1000 cells in stationary phase (*n *= 840 cells) and exponential phase (*n *= 878 cells).

**FIG 2:**
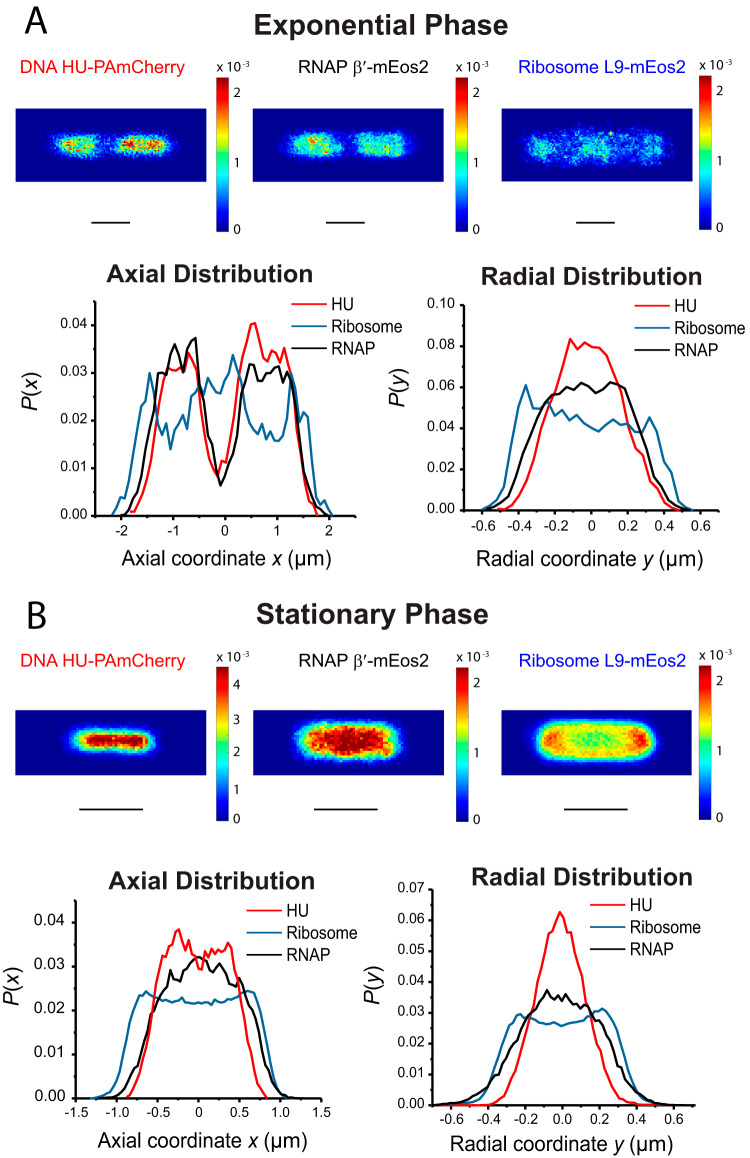
Cell-averaged superresolution spatial distributions of DNA (HU-PAmCherry), RNAP (β′-mEos2), and ribosome (L9-mEos2) in exponential phase and stationary phase. (A) Cells in exponential phase with lengths of 4.0 to 4.3 μm. (Top) 2D location heat map of DNA (left), RNAP (middle), and ribosomes (right). Pixel size, 40 nm by 40 nm. Color scale indicates probability per pixel; sum over all pixels equals 1. (Bottom) Projected axial (left) and radial (right) distribution of HU (red), RNAP (green), and ribosome (blue). Radial distribution includes only molecules in the nucleoid region (0.4 μm < $\left|x\right|$ < 1.2 μm). (B) Cells in stationary phase with length of 2.3 to 2.5 μm. (Top) 2D heat maps of DNA (left), RNAP (middle), and ribosome (right) spatial distributions. Pixel size, 40 nm by 40 nm. Color scale indicates probability per pixel; sum over all pixels equals 1. (Bottom) Axial (left) and radial (right) distribution of HU (red), RNAP (green), and ribosome (blue). Radial distribution includes only molecules in the nucleoid region ($\left|x\right|$ < 0.5 μm).

We begin with a qualitative comparison of overall cell and nucleoid morphology in fairly rapid exponential growth (45 min doubling time at 30°C) versus 2-day stationary phase. The strains used in this study are described in Table 1. In Fig. 1, phase-contrast images of a field of VH1000 (WT) cells with nucleoids stained by Sytox Green are compared for exponential growth (Fig. 1A) and 2-day stationary phase (Fig. 1B). Cell length was estimated as the tip-to-tip length of the cell mesh provided by the program Oufti (32). The distribution of cell lengths obtained by combining many fields of view is shown in Fig. 1C. Length distributions for the other strains in stationary phase are similar (see Fig. S2A in the supplemental material). The WT cells are much shorter in 2-day stationary phase than in exponential phase. The mean cell length in 2-day stationary phase is 2.6 ± 0.5 μm (± 1 standard deviation). This compares favorably with two previous results: 2.7 ± 0.2 μm for an overnight culture (22) and ∼2.2 μm for a 1-day stationary-phase culture (see Fig. S1 in reference 23). All cells remain spherocylindrical in stationary phase; 10% have aspect ratios of <2.0 (Fig. S2C). Cell widths determined from Oufti are compared with transverse peak-to-peak distances from images of FM4-64 membrane dye in Fig. S2B. Both methods indicate that cells were significantly narrower in stationary phase than in exponential phase. In stationary phase, the nucleoids were also shorter in length, as qualitatively shown by the overlaid fluorescence images of DNA stained by Sytox Green. In exponential phase, there were 2 to 4 nucleoid lobes for most cells. In stationary phase, most cells show one compact nucleoid lobe, but about 20% of cells show two lobes. The following sections provide a quantitative analysis of the data.

**TABLE 1:** Bacterial strains used in this study

Strain	Species imaged	Reference(s) (source[s] of strain details)	Background strain	Expression method
JCW154	*Right2* by *parS*, ParB-GFP	38	MG1655	Plasmid
VH1000	WT		VH1000	
SM7	HU-PAmCherry	31	VH1000	Plasmid
MSG196	Ribosome S2-mEos2	52	VH1000	Chromosome
SB1	Ribosome L9-mEos2	22, 52	VH1000	Chromosome
HC1	RNAP β′-mEos2	33	VH1000	Chromosome
JCW96	Kaede	45	VH1000	Plasmid

10.1128/mBio.00143-20.3 FIG S2 (A) Cell length distributions from phase-contrast images of different strains in 2-day stationary phase. (B) Correlation of cell widths as determined by two different methods that examined (i) the Oufti mesh drawn for phase-contrast images and (ii) the distance between the two peaks of a transverse line scan through a fluorescence image of the cell outline from membrane binding dye FM4-64. The vertical lines mark the mean width of single-cell Kaede distributions, taken to be twice the best-fit radius to the cylindrical model. The resulting Kaede width values were 0.82 ± 0.04 μm in exponential phase and 0.50 ± 0.12 μm in stationary phase. It is plausible that Kaede fills the cytoplasm in exponential phase, but its distribution is much narrower than the cytoplasm in stationary phase. (C) Distributions of aspect ratios (length/width from Oufti cell outlines derived from phase-contrast images) in stationary phase and exponential growth (47-min doubling time). Download FIG S2, EPS file, 1.6 MB. Copyright © 2020 Zhu et al. 2020 Zhu et al. This content is distributed under the terms of the Creative Commons Attribution 4.0 International license.

### Projected 2D spatial distributions of DNA, RNAP, and ribosomes.

The microscope projects fluorescence from single molecules within the 3D cell onto the 2D camera plane. Here, *x* is the coordinate along the main cell axis, *y* is the coordinate along the shorter, transverse axis, and *z* is the vertical axis of the microscope. The superresolution location measurements provide cell-averaged, projected 2D spatial distributions by pooling accurate single-molecule location data (*x*, *y*) from many individual protein copies within many cells. These spatial distributions provide quantitative insights into the size of the nucleoid and which species are able to penetrate its interior.

In order to minimize heterogeneity within each averaged spatial distribution, we focus the analysis on data from a narrow range of cell lengths near the peak of each length distribution (Fig. 1C). For the exponential-phase data, we used the range 4.0 to 4.3 μm; for the stationary-phase data, we used 2.3 to 2.5 μm. In Fig. 2, we compare spatial distributions of DNA (HU-PAmCherry labeling), RNAP (β′-mEos2), and ribosomal species (L9-mEos2), averaged across many cells within the chosen length range, with all three species imaged at 30 ms/frame.

In exponential phase (Fig. 2A), the microscope-projected 2D location heat maps indicate a large degree of spatial overlapping of the averaged DNA and RNAP distributions. Neither distribution fills the entire cytoplasm. Such sequestration of RNAP within the nucleoid arises from frequent specific and nonspecific binding events to the chromosomal DNA (33). These are strong enough and frequent enough to keep RNAP within the nucleoid or nearby. Each axial distribution comprises two well-separated lobes. In contrast, the ribosome distribution exhibits three “ribosome-rich regions” that are strongly segregated from the nucleoids. For a more quantitative view, we projected each 2D distribution onto the long cell axis (*x*) and onto the short cell axis (*y*) to form 1D axial and “radial” distributions, *P*(*x*) and *P*(*y*). The 1D radial distributions for DNA, RNAP, and ribosomes include only those molecules found in the two axial regions (0.4 μm < $\left|x\right|$ < 1.2 μm) where the two nucleoid lobes lie. This range includes 39% of all ribosomal species, 63% of all RNAP copies, and 64% of all HU copies. These selective radial distributions provide the clearest view of whether ribosomes and RNAP tend to concentrate inside or outside the nucleoids (or neither). In exponential phase, the full width at half maximum height (FWHM) of the DNA radial distribution is 470 nm. Comparing DNA and RNAP, the axial distributions overlap strongly; the radial distribution of RNAP is wider (650 nm FWHM) than that of DNA. Comparing DNA and ribosomes, both axial and radial 1D distributions show strong anticorrelation. In other words, the ribosomes are concentrated axially in the endcaps and at cell center and radially in the thin annular region surrounding each nucleoid lobe. These results are consistent with previous work using S2-mEos2 labeling (34) and S2-YFP (S2-yellow fluorescent protein) labeling (35, 36).

In stationary phase, even among cells in the narrow length range of 2.3 to 2.5 μm, we observe clear cell-to-cell heterogeneity of the DNA spatial distributions. About 20% of these cells have two nucleoid lobes, while 80% have one compact nucleoid (Fig. S3). Cells are judged to have two nucleoid lobes if the peak-to-valley ratio in the axial intensity distribution is >1.5. The fraction of cells having two lobes increases with increasing cell length (Fig. S3B). Combining superresolution localization data from all cells in the 2.3 to 2.5 μm range, Fig. 2B displays 2D heat maps and 1D projected axial and radial distributions for DNA, RNAP, and ribosomes in stationary phase. The radial profiles exclude the end cap regions and utilize only those molecules in the nucleoid region ($\left|x\right|$ < 0.5 μm). This region includes 55% of all ribosomal species, 71% of all RNAP copies, and 83% of all HU copies. The averaged DNA radial distribution *P*(*y*) is substantially narrower in stationary-phase cells than in exponential-phase cells (FWHM of 300 nm versus 470 nm). The averaged DNA axial distribution in stationary phase is similar in length to that of a single nucleoid lobe in exponential phase (1,120 nm FWHM versus 1,080 nm FWHM). As in exponential phase, in stationary phase the DNA and RNAP distributions show strong spatial overlap. The RNAP distribution is slightly longer and substantially wider (540 nm FWHM) than the DNA distribution (300 nm FWHM). The single-peaked RNAP radial distribution demonstrates that RNAP can penetrate the stationary-phase nucleoid. If RNAP were strongly excluded from the nucleoid, we would observe a double-peaked radial distribution like that of the ribosomes (which concentrate in the annular region around the nucleoid). This is consistent with a recent report (23) of transcriptional activity in stationary-phase cells. The ratio of RNAP to DNA FWHMs from the radial distributions is higher in stationary phase (540 nm/300 nm = 1.80) than in exponential phase (650 nm/470 nm = 1.38). This suggests somewhat less effective sequestration of RNAP within and near to the nucleoids of stationary-phase cells, consistent with weaker and/or less frequent binding events. The RNAP diffusion results corroborate this idea.

10.1128/mBio.00143-20.4FIG S3(A and B) Examples of single-cell DNA (HU-PAmCherry) spatial distributions exhibiting one axial lobe (A) or two axial lobes (B). (Top) Scatter plot of HU locations. Red line is cell mesh generated from phase-contrast image using Oufti program. (Middle) Axial distribution of HU locations. (Bottom) Radial distribution of HU locations. Each radial distribution includes only molecules in the nucleoid region ($\left|x\right|$ < 0.5 μm for one-lobed cell and 0.2 μm < $\left|x\right|$ < 0.6 μm for two-lobed cell). The black line represents a simulated radial projection of particles uniformly distributed within a spherocylinder of radius *r*_1−cell_ = 0.20 μm. (C) Number of cells with one or two axial HU lobes as a function of cell length. (D) Number of cells with one or two DNA loci determined by counting *Right2* puncta as a function of cell length. Download FIG S3, PDF file, 1.3 MB.Copyright © 2020 Zhu et al.2020Zhu et al.This content is distributed under the terms of the Creative Commons Attribution 4.0 International license.

### Subdiffusive motion of DNA.

The chromosomal DNA is a long, circular biopolymer confined to a volume much smaller than its natural size by the E. coli cell envelope. On a timescale of ∼100 s, displacement of small segments of the polymer is constrained by attachment within the polymer chain. Instead of free diffusion, the local motion is a type of subdiffusion, which we and others call “jiggling” (37).

**FIG 3:**
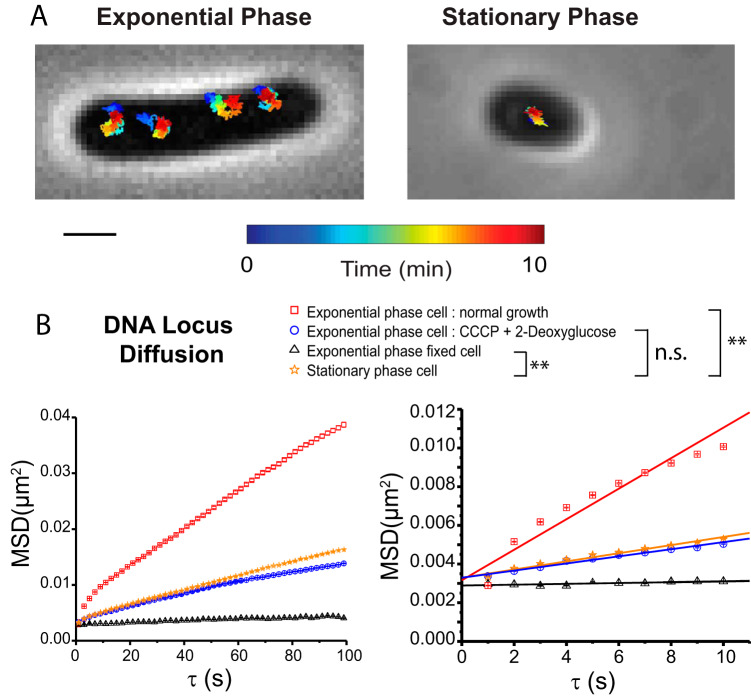
(A) Time lapse trajectories of DNA *Right2* loci over 10 min at 12 s per frame in exponential phase (left) and stationary phase (right). Time is color-coded as shown. Trajectories are superimposed on phase-contrast image. Scale bar: 1 μm. (B) Mean-square displacement (MSD) versus lag time for DNA loci under conditions of various treatments, obtained from movies taken at 1 s/camera frame with 600 frames/movie. Each point on each curve represents the mean of approximately 30,000 to 100,000 individual step lengths. (Left) MSD covering 100 s; only half the data are plotted for clarity. (Right) expanded view of the first 10 s. Data representing apparent diffusion coefficient *D*_app_ were obtained from linear fit to the first 10 points. Numerical results are as follows: *D*_app_ = (2.0 ± 0.2) × 10*^−^*^4^ μm^2^/s for normal growth in exponential phase; (4.6 ± 0.2) × 10*^−^*^5^ μm^2^/s after CCCP treatment; (7.6 ± 2.1) × 10*^−^*^6^ μm^2^/s for fixed cells; and (5.1 ± 0.3) × 10*^−^*^5^ μm^2^/s for cells in 48-h stationary phase. Statistical testing (see the supplemental material) showed that the *D*_app_ value determined for cells in stationary phase is significantly different from that determined for normal-growth, exponential-phase cells (*p* = 2.2 × 10*^−^*^6^) and from that determined for fixed cells (*p* = 3.6 × 10*^−^*^10^). Results from CCCP-treated cells were not significantly different from those from cells in stationary phase (*p* = 0.14).

To characterize the local dynamics of chromosomal DNA in both the exponential and stationary phases, we tracked the DNA locus *Right2* labeled by the fusion protein ParB-GFP (ParB-green fluorescent protein) (strain JCW154; Table 1) (34, 38, 39). At an imaging rate of 1 frame/s, we were able to monitor the locus position with an accuracy of σ ~ 30 nm over 600 frames = 10 min. Representative 10-min trajectories in the exponential and stationary phases are shown in Fig. 3A. In Fig. 3B, we compare plots of mean-square displacement versus lag time [MSD(τ)] for exponentially growing untreated cells, for such cells treated with carbonyl cyanide *m*-chlorophenylhydrazone (CCCP) plus 2-deoxy-glucose (which depletes ATP by dissipation of the proton motive force and prevention of glycolysis) (40), for such cells after chemical fixation by formaldehyde (34), and for untreated cells in 2-day stationary phase. Each plot includes data obtained from the entire distribution of cell lengths. The plots are displayed on two timescales: 0 to 100 s and 0 to 10 s.

These MSD plots exhibit negative curvature, the signature of subdiffusive motion, as expected for the local dynamics of a small segment of a large, confined polymer. The degrees of curvature differ across the different cases, especially over the first 10 s. To enable semiquantitative comparisons on the 10-s timescale, for each MSD curve we computed an apparent diffusion coefficient *D*_app_ from the slope of the linear least-squares fit to the first 10 experimental points. The results are as follows: *D*_app_ = (2.0 ± 0.2) × 10^−4^ μm^2^/s for cells in normal exponential growth; *D*_app_ = (4.6 ± 0.2) × 10^−5^ μm^2^/s after CCCP treatment; and *D*_app_ = (7.6 ± 2.1) × 10^−6^ μm^2^/s for fixed cells (34). In stationary phase, *D*_app_ = (5.1 ± 0.3) × 10*^−5^* μm^2^/s, representing a value four times lower than that determined for untreated cells in exponential phase and comparable to that determined for exponential-phase cells following treatment with CCCP. We performed two-tailed Student’s *t* tests for statistically significant differences between values of *D*_app_ obtained for stationary-phase cells and for cells maintained under other experimental conditions (see Text S1 and Fig. S4 and S5 in the supplemental materials). In this work, we use the significance level α = 0.01 throughout. Significance results are included in the figure captions. The value representing *D*_app_ for the jiggling motion of DNA was found to be significantly higher for the cells in the normal, exponential-growth phase than for the stationary-phase cells (Fig. 3).

10.1128/mBio.00143-20.1 TEXT S1 Image analysis for tracking, statistical test for determination of significant differences in MSD slopes, and Monte Carlo simulations to fit experimental *P*(*r*) distributions. Download Text S1, DOCX file, 0.1 MB. Copyright © 2020 Zhu et al. 2020 Zhu et al. This content is distributed under the terms of the Creative Commons Attribution 4.0 International license.

We also carried out single-molecule tracking experiments at 30 ms/frame using the nonspecific DNA-binding protein HU labeled by the photoactivatable fluorescent protein PAmCherry (strain SM7; Table 1). These trajectories presumably involve an average over time spent diffusing “freely” (not bound to DNA) within the nucleoid and time spent specifically or nonspecifically bound to DNA. The corresponding MSD plots (Fig. S4) are quite linear on a 180-ms timescale. In this case, we estimate an apparent diffusion coefficient from the slope of the linear least-squares fit to the first three experimental points. The numerical results are *D*_app_ = 0.098 ± 0.004 μm^2^/s in exponential phase and *D*_app_ = 0.088 ± 0.004 μm^2^/s in stationary phase. The *t* test with α = 0.01 showed no significant difference in *D*_app_ values between the two cases (Fig. S4).

10.1128/mBio.00143-20.5 FIG S4 Cell-averaged HU-PAmCherry MSD versus lag time plots from movies taken at 30 ms/frame for exponential-phase and stationary-phase cells. Linear fitting of the first three data points yields the apparent apparent diffusion coefficient *D*_app_. The numerical results were as follows: *D*_app_ = 0.098 ± 0.004 μm^2^/s in exponential phase and *D*_app_ = 0.088 ± 0.004 μm^2^/s in stationary phase. The localization error values (σ) were 54 nm in exponential phase and 51 nm in stationary phase. For a linear least-squares fit of the data up to τ = 6 steps, *R*^2^ = 0.998 for exponential phase and *R*^2^ = 0.997 for stationary phase. Statistical testing (see above) showed that the two values of *D*_app_ were not statistically significantly different (*p* = 0.26). Download FIG S4, EPS file, 1.4 MB. Copyright © 2020 Zhu et al. 2020 Zhu et al. This content is distributed under the terms of the Creative Commons Attribution 4.0 International license.

### Diffusion of RNAP (β′-mEos2) in stationary phase.

**FIG 4:**
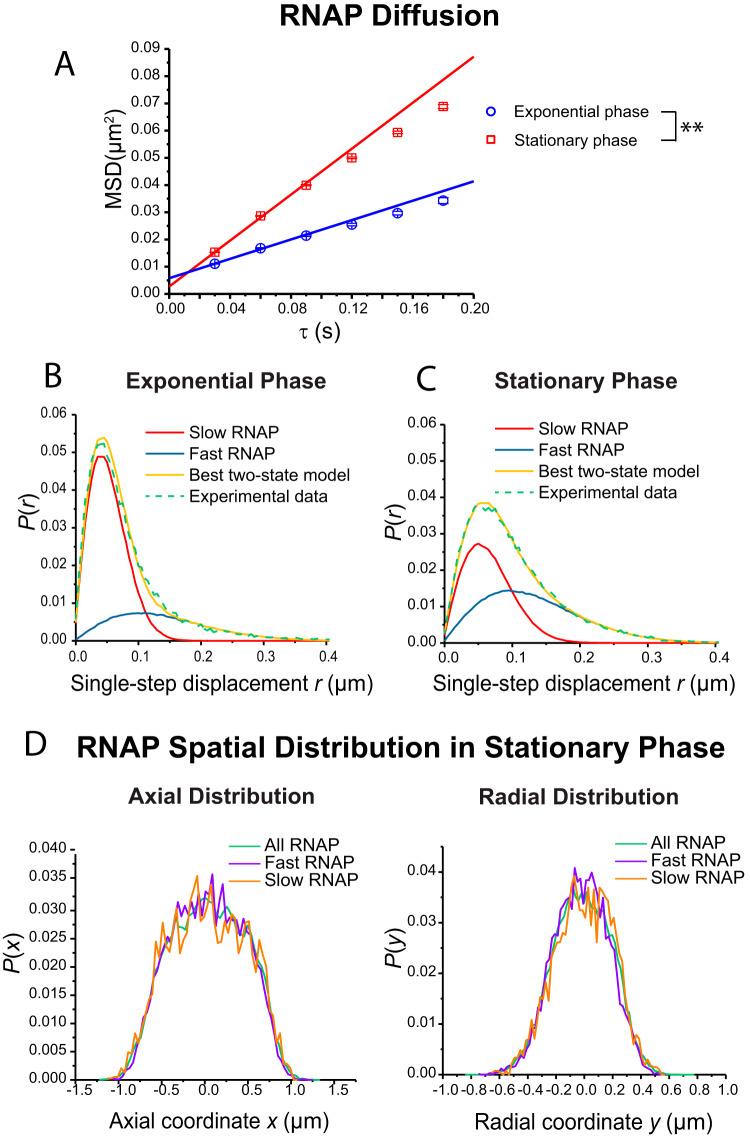
(A) RNAP β*′*-mEos2 mean-square displacement versus lag time τ for exponential-phase and stationary-phase cells. Movies were acquired at 30 ms per frame. Data representing apparent diffusion coefficient *D*_app_ values were obtained by linear fitting of the first three data points. Numerical results are *D*_app_ = 0.043 ± 0.003 μm^2^/s for exponential phase and *D*_app_ = 0.103 ± 0.005 μm^2^/s for stationary phase. Localization error σ is 44 nm in exponential phase and 43 nm in stationary phase. For both cases, *R*^2^ = 0.994 for a linear least-squares fit of data to τ = 6 steps. Statistical testing (see the supplemental material) found that the *D*_app_ values for RNAP in exponential phase and in stationary phase were significantly different from each other (*p* = 0.008). (B) RNAP single-step displacements in exponential phase from β*′*-mEos2 trajectories at 30 ms per frame. Green, experimental data; yellow, best fit to static two-state model. Model parameters: *D*_slow_ = 0.033 μm^2^/s (σ_slow_ = 20 nm), *f*_slow_ = 0.72, *D*_fast_ = 0.30 μm^2^/s (σ_fast_ = 50 nm), *f*_fast_ = 0.28. Slow and fast components are as labeled. (C) Data for RNAP in stationary-phase cells acquired and analyzed as in panel B. Best-fit model parameters: *D*_slow_ = 0.034 μm^2^/s (σ_slow_ = 30 nm), *f*_slow_ = 0.49, *D*_fast_ = 0.24 μm^2^/s (σ_fast_ = 50 nm), *f*_fast_ = 0.51. (D) Axial (left) and radial (right) distribution of rapidly diffusing RNAP (single-step displacement *r *> 0.15 μm) and slowly diffusing RNAP (single-step displacement *r *< 0.03 μm). Data representing the distribution of all the RNAP (Fig. 2B) are shown for comparison. Radial distributions include only molecules in the nucleoid region ($\left|x\right|$ < 0.5 μm).

Next, we used single-particle tracking to compare data representing RNAP diffusion in stationary phase versus exponential phase. We tracked RNAP (β′-mEos2) diffusion with superresolution imaging at 30 ms/frame using strain HC1 (Table 1). The mass of a complete RNAP (including the σ subunit) is ∼450 kDa. Using data from all cells regardless of length, we analyzed only those trajectories that lasted 6 steps or longer and truncated the longer trajectories to 6 steps. The MSD plots for RNAP (Fig. 4A) are fairly linear over lag times of up to 180 ms. We estimated an apparent diffusion coefficient from the slope of the first three data points, yielding *D*_app_ = 0.043 ± 0.003 μm^2^/s in exponential phase and *D*_app_ = 0.103 ± 0.005 μm^2^/s in stationary phase. The *t* test with α = 0.01 showed that this difference was statistically significant (Fig. 4). Surprisingly, the population-weighted mean apparent diffusion coefficient for RNAP in stationary phase was 2-fold higher than in exponential growth.

The same trajectories can be divided into individual steps of 30-ms duration to provide the distribution of single-step displacements *P*(*r*) in exponential growth and in stationary phase (Fig. 4B and C). We modeled *P*(*r*) distributions in each case using a two-state model which included confinement effects and localization uncertainty. This “static” model assumes that the displacements arise from a combination of a slowly diffusing population and a rapidly diffusing population that do not exchange on the 30-ms imaging timescale. The numerical least-squares procedure is described in more detail in Materials and Methods and in earlier work (31, 41, 42). The fitting parameters represent the diffusion coefficients of the slow and fast populations, *D*_slow_ and *D*_fast_, and the fraction of slow copies *f*_slow_; by subtraction, *f*_fast_ = 1 – *f*_slow_.

In exponential phase, the best fit to *P*(*r*) gave the parameters *D*_slow_ = 0.033 μm^2^/s, *f*_slow_ = 0.72, *D*_fast_ = 0.30 μm^2^/s, and *f*_fast_ = 0.28. As suggested before (33), on the 30-ms timescale of single steps, the slow population likely represents DNA-bound RNAP (both transcribing RNAP and RNAP bound nonspecifically to DNA). The fast population likely represents RNAP undergoing free diffusion, primarily within the nucleoid volume. In stationary phase, the best fit to *P*(*r*) gave the parameters *D*_slow_ = 0.034 μm^2^/s, *f*_slow_ = 0.49, *D*_fast_ = 0.24 μm^2^/s, and *f*_fast_ = 0.51.

Comparing stationary phase to exponential phase, we see that the coefficients representing fast and slow diffusion remained reasonably constant. The primary difference is the larger fraction of rapidly diffusing RNAP in stationary phase. In stationary phase, the rapidly diffusing and slowly diffusing RNAP copies show very similar spatial distributions (Fig. 4D). Taken together, these results suggest that RNAP in stationary phase spends a smaller fraction of time bound to DNA but that the binding events still remain strong enough and frequent enough to sequester the RNAP within a volume only moderately larger than the nucleoid volume. The structure of the DNA on the nanometer length scale may also be different, as discussed below.

### Diffusion of ribosomal species in stationary phase.

We tracked diffusion of ribosomal species using two different strains, MSG196 (S2-mEos2 labeling) and SB1 (L9-mEos2), in both exponential phase and stationary phase (Table 1). The ribosome movies were acquired at 30 ms/frame. We focus on the data from the L9-labeled strain because it likely enables 100S dimers to achieve their natural binding conformation. The S2 protein sits at the interface between monomer 70S units of the 100S homodimer (43); mEos2 labeling of S2 likely interferes with formation of this structure (44). The MSD plots for S2-mEos2 labeling of 30S and L9-mEos2 labeling of 50S are compared in Fig. S5. In exponential growth, the two labeling schemes produced quantitatively similar MSD plots. There is no statistically significant difference. In stationary phase, however, the population-weighted mean ribosome diffusion coefficient from the MSD plot was almost twice as large for S2 labeling (Fig. S5B), and this difference was found to be statistically significant. This is consistent with reduced formation of bulky 100S particles in the cells with S2 labeling. In stationary phase, the nucleoid morphologies and degrees of DNA-ribosome segregation were similar for the two labeling schemes (Fig. S5A and C).

10.1128/mBio.00143-20.6FIG S5Spatial distribution and mean diffusion of ribosomal species labeled with the S2-mEos2 and L9-mEos2 constructs. (A) 2D location heat map representing data averaged across cells in the length range 2.3 to 2.5 μm in stationary phase. Pixel dimensions are 40 nm by 40 nm. The color scale represents probability per pixel, normalized so that sum is 1. Scale bar: 1 μm. (B) MSD versus lag time τ from trajectories taken at 30 ms per frame in four different cases: L9 and S2 labeling in both exponential growth and 2-day stationary phase. The apparent diffusion coefficient *D*_app_ was obtained by linear fitting of the first three data points. The numerical results are as follows: *D*_app_ = 0.035 ± 0.001 μm^2^/s for L9 labeling in exponential growth; *D*_app_ = 0.080 ± 0.002 μm^2^/s for L9 labeling in stationary phase; *D*_app_ = 0.042 ± 0.001 μm^2^/s for S2 labeling in exponential growth; and *D*_app_ = 0.151 ± 0.002 μm^2^/s for S2 labeling in stationary phase. Estimated localization error values are 32 nm, 41 nm, 34 nm, and 47 nm, respectively. For a linear least-squares fit of the data up to τ = 6 steps, *R*^2^ values are 0.997, 0.998, 0.999, and 0.998, respectively. Exponential-phase data representing S2 labeling are from reference 11. In stationary phase, the *D*_app_ value determined for cells labeled by S2-mEos2 was almost twice that determined for cells labeled by L9-mEos2. Statistical testing (see above) found no significant difference between S2 labeling and L9 labeling in exponential phase (*p* = 0.21). Differences between S2 and L9 labeling in stationary phase were statistically significant (*p* = 0.007). Differences between S2 labeling in stationary phase versus exponential phase were also statistically significant (*p* = 0.002). (C) Axial (left) and radial (right) distributions of ribosome in stationary-phase cells with length of 2.3 to 2.5 μm. Radial distribution data include only results representing molecules in the nucleoid region ($\left|x\right|$ < 0.5 μm). Download FIG S5, EPS file, 1.6 MB.Copyright © 2020 Zhu et al.2020Zhu et al.This content is distributed under the terms of the Creative Commons Attribution 4.0 International license.

**FIG 5:**
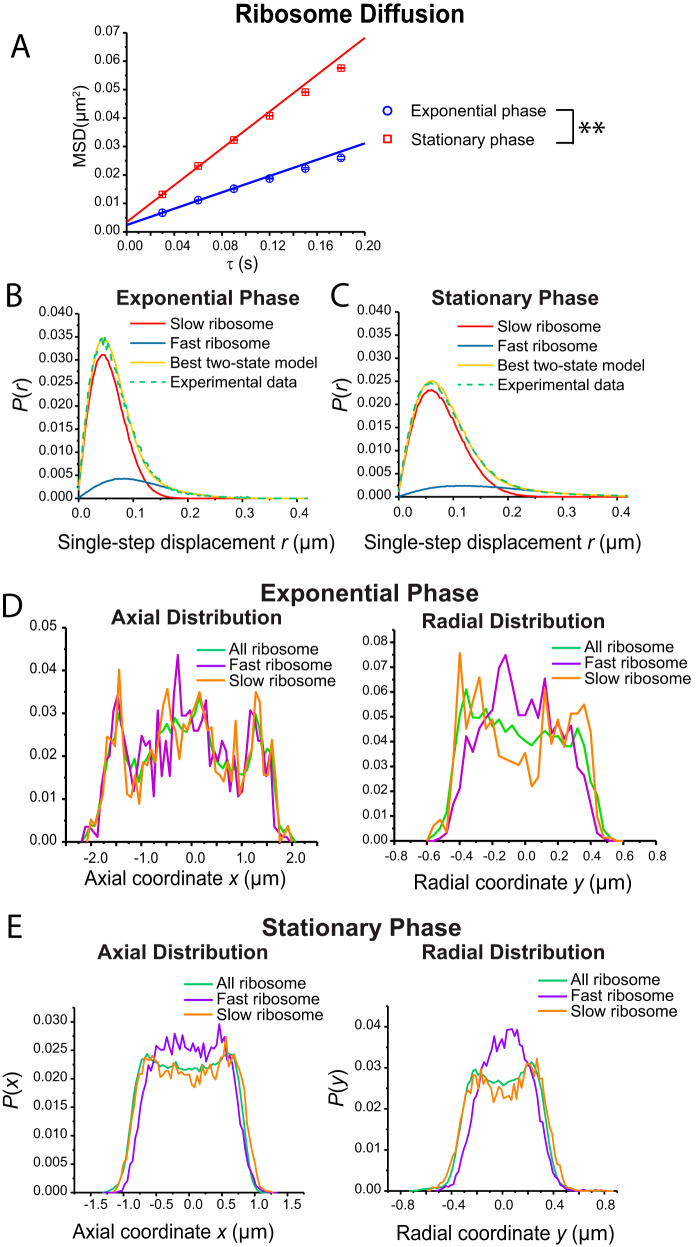
(A) Ribosome L9-mEos2 mean-square displacement versus lag time τ from trajectories taken at 30 ms per frame in exponential phase and stationary phase. Data representing apparent diffusion coefficient *D*_app_ were obtained by linear fitting of the first three data points. Numerical results: *D*_app_ = 0.035 ± 0.001 μm^2^/s for exponential phase and *D*_app_ = 0.080 ± 0.002 μm^2^/s for stationary phase. Estimated localization errors σ are 32 nm and 41 nm, respectively. For a linear least-squares fit of the data up to τ = 6 steps, *R*^2^ = 0.997 for exponential phase and *R*^2^ = 0.998 for stationary phase. Statistical testing (see the supplemental material) found that *D*_app_ values for L9-mEos2 in exponential phase and in stationary phase are significantly different from each other (*p* = 0.003). (B) Ribosome single-step displacements in exponential phase from L9-mEos2 trajectories at 30 ms per frame. Green, experimental data; yellow, best fit to a static two-state model. Slow and fast components are as labeled. Model parameters: *D*_slow_ = 0.025 μm^2^/s (σ_slow_ = 25 nm), *f*_slow_ = 0.80, *D*_fast_ = 0.16 μm^2^/s (σ_fast_ = 40 nm), *f*_fast_ = 0.20. (C) Data for ribosomes in stationary-phase cells acquired and analyzed as in panel B. Best-fit model parameters: *D*_slow_ = 0.084 μm^2^/s (σ_slow_ = 25 nm), *f*_slow_ = 0.82, *D*_fast_ = 0.60 μm^2^/s (σ_fast_ = 43 nm), *f*_fast_ = 0.18. (D) For exponential-phase cells, axial (left) and radial (right) distribution of rapidly diffusing ribosome copies (fastest 10% population, single-step displacement *r *> 0.120 μm) and slowly diffusing ribosome copies (slowest 10% population, single-step displacement *r *< 0.021 μm). Data representing the distribution of all ribosomes (Fig. 2B) are shown for comparison. Radial distributions include only molecules in the nucleoid region (0.4 μm *<* $\left|x\right|$ *<* 1.2 μm). (E) For stationary-phase cells, axial (left) and radial (right) distribution of rapidly diffusing ribosome copies (fastest 10% population, single-step displacement *r *> 0.171 μm) and slowly diffusing ribosome copies (slowest 10% population, single-step displacement *r *< 0.027 μm). Data representing the distribution of all ribosomes (Fig. 2B) are shown for comparison. Radial distributions include only molecules in the nucleoid region ($\left|x\right|$ < 0.5 μm).

Focusing now on L9-mEos2 labeling (Fig. 5), we compare MSD plots, single-step displacement distributions, and projected 1D axial and radial distributions for ribosomal species in exponential phase and stationary phase. The apparent diffusion coefficient *D*_app_ is obtained by linear least-squares fitting of the first three data points. The numerical results are as follows: *D*_app_ = 0.035 ± 0.001 μm^2^/s for exponential phase and *D*_app_ = 0.080 ± 0.002 μm^2^/s for stationary phase. The difference is statistically significant. Averaged across whatever L9-containing species may be present in each case, the population-weighted mean diffusion coefficient is twice as high in stationary phase.

Next, we analyzed the ribosome single-step displacement distributions *P*(*r*) using the same static, two-state numerical model (Fig. 5B and C). In exponential phase, the best fit to *P*(*r*) gave the parameters *D*_slow_ = 0.025 μm^2^/s, *f*_slow_ = 0.80, *D*_fast_ = 0.16 μm^2^/s, and *f*_fast_ = 0.20. In stationary phase, the best fit to *P*(*r*) gave the parameters *D*_slow_ = 0.084 μm^2^/s, *f*_slow_ = 0.82, *D*_fast_ = 0.60 μm^2^/s, and *f*_fast_ = 0.18. Both *D*_slow_ and *D*_fast_ are much higher in stationary phase, while the relative populations remain similar.

The projected axial and radial distributions in exponential phase and stationary phase are compared in Fig. 5D and E. In both growth phases, the distributions are qualitatively similar in the sense that the ribosomal species tend to concentrate in ribosome-rich regions outside the nucleoid(s). This is true both axially and radially. The main difference is the presence of two nucleoid lobes in exponential growth versus only one in stationary phase. We also plotted separate distributions for the 10% fastest and 10% slowest copies; these distributions are noisier because they include fewer molecules. In both growth phases, the fastest-diffusing species tended to concentrate within the nucleoids whereas the slowest-diffusing species were those most extensively segregated from the nucleoids. We discuss these results in more detail below.

### Spatial distributions and diffusive properties of Kaede.

**FIG 6:**
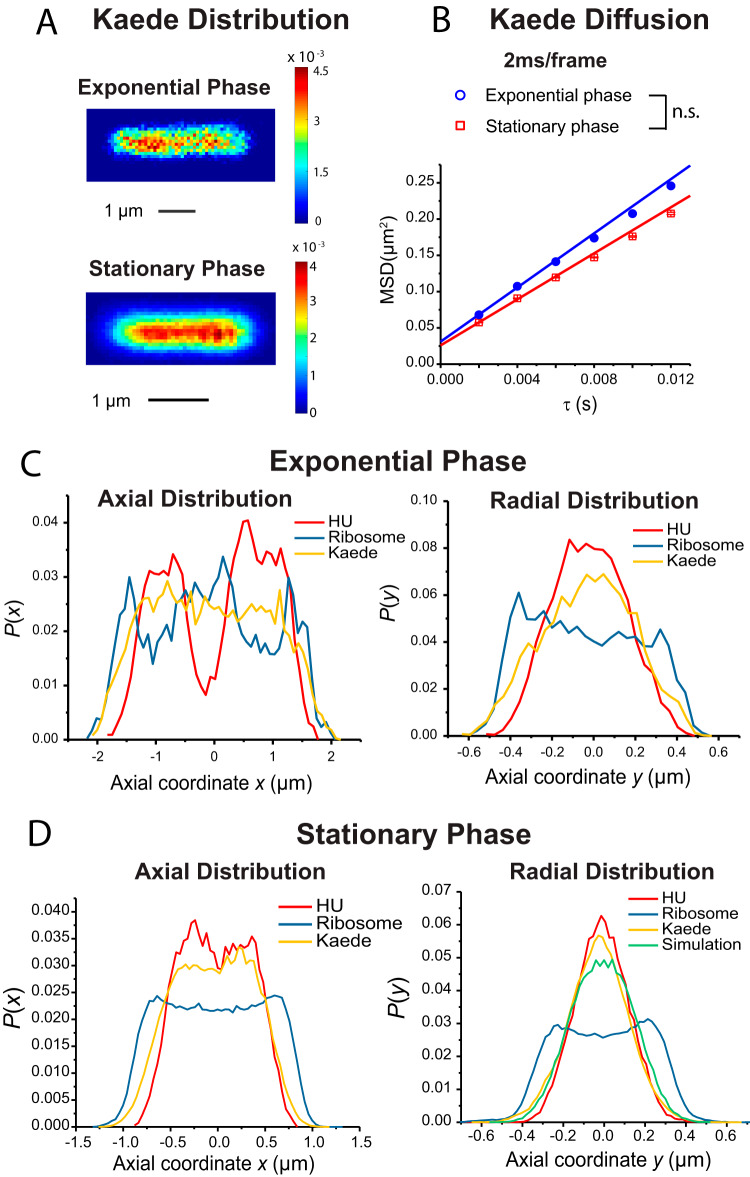
Kaede spatial distributions and diffusion dynamics. (A) 2D heat maps of Kaede spatial distributions averaged across cells in the length range 4.0 to 4.3 μm in exponential phase (top) and 2.3 to 2.5 μm in stationary phase (bottom). Pixel size, 80 nm by 80 nm for exponential phase and 40 nm by 40 nm for stationary phase. Color scale indicates probability per pixel; sum over all pixels equals 1. (B) Kaede MSD versus lag time from trajectories taken at 2 ms per frame in exponential phase and stationary phase. Data representing apparent diffusion coefficient *D*_app_ were obtained by linear fitting of the first three data points. Numerical results are as follows: *D*_app_ = 4.58 ± 0.19 μm^2^/s for exponential phase in fast growth and *D*_app_ = 3.88 ± 0.17 μm^2^/s for stationary phase. Estimated localization error σ is 105 nm and 97 nm, respectively. For both cases, *R*^2^ = 0.998 in a linear least-squares fit of the data up to τ = 6 steps. Statistical testing (see the supplemental material) found that *D*_app_ for Kaede in normal growth exponential phase and in stationary phase are not significantly different from each other (*p* = 0.115). (C) Axial (left) and radial (right) distribution of HU (red), ribosomes (blue), and Kaede (yellow) averaged across exponential-phase cells in the length range 4.0 to 4.3 μm. Radial distribution includes only molecules in the nucleoid region (0.4 μm < $\left|x\right|$ < 1.2 μm). HU and ribosome data were replotted from Fig. 2A for comparison. (D) Data were determined as described for panel C but were averaged for stationary-phase cells in the length range 2.3 to 2.5 μm. Radial distribution includes only molecules in the nucleoid ($\left|x\right|$ < 0.5 μm). HU and ribosome data from Fig. 2B were replotted for comparison. The green line represents a calculated projection of a radial distribution of constant density within a cylinder of radius 250 nm. The simulation used *D*_app_ = 3.88 μm^2^/s and localization error σ = 97 nm derived from panel B.

Here, we explored the behavior of a soluble, exogeneous protein in the stationary-phase cytoplasm. Kaede is a photoconvertible fluorescent protein believed to occur in the bacterial cytoplasm as a tetramer of total mass 110 kDa (45). Kaede is expressed from a plasmid in strain JCW96 (Table 1). To minimize blurring due to very fast diffusion, we imaged Kaede at 2 ms/frame. The Kaede localization heat maps averaged across cells for exponential phase and for stationary phase are compared in Fig. 6A. In each case, the range of cell lengths included in the analysis was the same as before. The Kaede MSD plots (Fig. 6B) yielded the following population-weighted mean diffusion coefficients: *D*_app_ = 4.58 ± 0.19 μm^2^/s for exponential phase and *D*_app_ = 3.88 ± 0.17 μm^2^/s for stationary phase. The *t* test with α = 0.01 indicated that these are not significantly different from each other (*p* = 0.115).

For exponential phase, 1D axial and radial projections of the cell-averaged Kaede spatial distributions are shown in Fig. 6C. Corresponding ribosome (L9-mEos2) and DNA (HU-PAmcherry) averaged distributions are included for comparison. In agreement with previous work, in exponential phase, Kaede distributes essentially uniformly throughout the cytoplasm (31, 45). There is no evidence of strong preferential interactions between Kaede and cytoplasmic components within the nucleoids compared with the ribosome-rich regions.

In stationary phase, analogous 1D axial and radial projections averaged across cells in the 2.3 to 2.5 μm length range are shown in Fig. 6D, which includes DNA and ribosome averaged distributions for comparison. Now the Kaede distribution is similar to that of DNA (HU-PAmCherry) and is very different from the ribosomal distribution. The situation is complicated by the cell-to-cell heterogeneity in the Kaede axial distributions. Within the narrow cell length range of 2.3 to 2.5 μm, about 80% of cells exhibited an axial distribution with a single peak at cell center. The remaining 20% exhibited an axial distribution with two peaks and a dip at cell center (peak/valley ratio of >1.5; Fig. S6). It is tempting to suggest that the 20% of cells having two axial Kaede peaks are the same 20% of cells having two axial DNA peaks, but we have not demonstrated this. The strong segregation of Kaede from the ribosomes along with sequestering of Kaede within the nucleoids suggests either some affinity between Kaede and the nucleoid region or some exclusionary force between Kaede and the ribosome-rich regions.

10.1128/mBio.00143-20.7FIG S6One-lobe and two-lobe Kaede spatial distributions in single stationary-phase cells. (A) Example of a one-lobe Kaede cell. (Left) Scatter plot of Kaede locations. The red line represents cell mesh generated from a phase-contrast image using Oufti. (Middle and right) Axial and radial projections of Kaede distributions for this cell. The radial distribution includes only molecules in the nucleoid region ($\left|x\right|$ < 0.5 μm). The black line represents simulated radial projections of particles uniformly distributed within a spherocylinder of radius *r*_1−cell_ = 0.27 μm. (B) Example of a two-lobe Kaede cell. Scatter plot, axial distribution, and radial distribution as shown. Radial distribution data include only molecules in the nucleoid region ($\left|x\right|$ < 0.5 μm). (C) Number of cells with one or two Kaede axial peaks as a function of cell length. (D) Cell-averaged axial distributions for stationary-phase cells having one or two lobes of Kaede and for stationary-phase cells having one or two axial lobes of DNA (HU-PAmCherry), each plotted separately for comparison. (E) Kaede axial distribution averaged over cells in stationary phase for the specific length ranges shown. Axial distributions averaged over exponentially growing cells are included for comparison. Download FIG S6, PDF file, 1.0 MB.Copyright © 2020 Zhu et al.2020Zhu et al.This content is distributed under the terms of the Creative Commons Attribution 4.0 International license.

We also estimated the mean radius of the Kaede distribution in stationary phase by fitting each single-cell Kaede radial distribution to a model of a spherocylinder uniformly filled with diffusing Kaede particles, with the radius as an adjustable parameter (Fig. S6A). The best-fit radii are then averaged across cells to obtain the mean radius of <*r_Kaede_*> = 0.25 ± 0.06 μm. Twice this radius is 0.50 μm, significantly smaller than the mean peak-to-peak width of 0.77 ± 0.03 μm from FM4-64 widefield images (Fig. S2B). Again, this indicates that Kaede concentrates within the stationary-phase nucleoid and avoids the ribosome-rich annular region.

### Quantitative estimates of nucleoid volume and DNA density in stationary phase.

In their coarse-grained simulation of LacI diffusion within the E. coli nucleoid, Chow and Skolnick found that the globular protein moves within the dense meshwork of DNA strands by hopping from one confining hole in the meshwork to another nearby hole (24). This “gated diffusion” process is driven by thermal fluctuations in the relative position of adjacent DNA strands. Accordingly, one of several key parameters required to understand target searches within the nucleoid is the average DNA density, which can be expressed in units of bp/μm^3^. The simulation used a DNA density of 7.6 × 10^7^ bp/μm^3^. Under the assumption that superresolution spatial distributions of HU-PAmCherry provide a good proxy for the chromosomal DNA distribution (31), we can provide rough estimates of the nucleoid volume and of the DNA density in 2-day stationary-phase cells. We will compare these estimates with those derived from our previous study of E. coli growing exponentially but very slowly (SM6 strain, 147-min doubling time) (31). Like stationary-phase cells, the very slowly growing cells typically exhibit only one nucleoid lobe comprising ∼1 chromosome of DNA (31).

First we estimate the number of complete chromosomes per cell in our stationary phase conditions. As cells enter stationary phase, initiation of chromosome replication halts, but elongation continues until termination. This means that each 2-day stationary-phase cell contains an integral number of complete chromosomes, as elegantly demonstrated by flow cytometry (46). We can estimate the distribution of chromosome copies per cell by counting the number of fluorescent puncta in each of the cells used to track the DNA locus *Right2* (Fig. 3A). For 447 of these cells in 2-day stationary phase (including all cell lengths), we observe either one or two such puncta per cell. The resulting distribution of chromosomal copy numbers versus cell length is shown in Fig. S3D. For these 447 cells, we found 244 with one locus and 203 with two, consistent with the intensity ratios of the flow cytometry study (see Fig. 5 in reference 46). For the 75 cells falling in the length range 2.3 to 2.5 μm, 33 had one locus and 42 had two loci. On average, those cells have ∼1.56 chromosome/cell. Each E. coli chromosome comprises 4.6 × 10^6^ bp of DNA. On average, there are *N*_avg_ ∼7.2 × 10^6^ bp of DNA per stationary-phase cell in the chosen length range.

**FIG 7:**
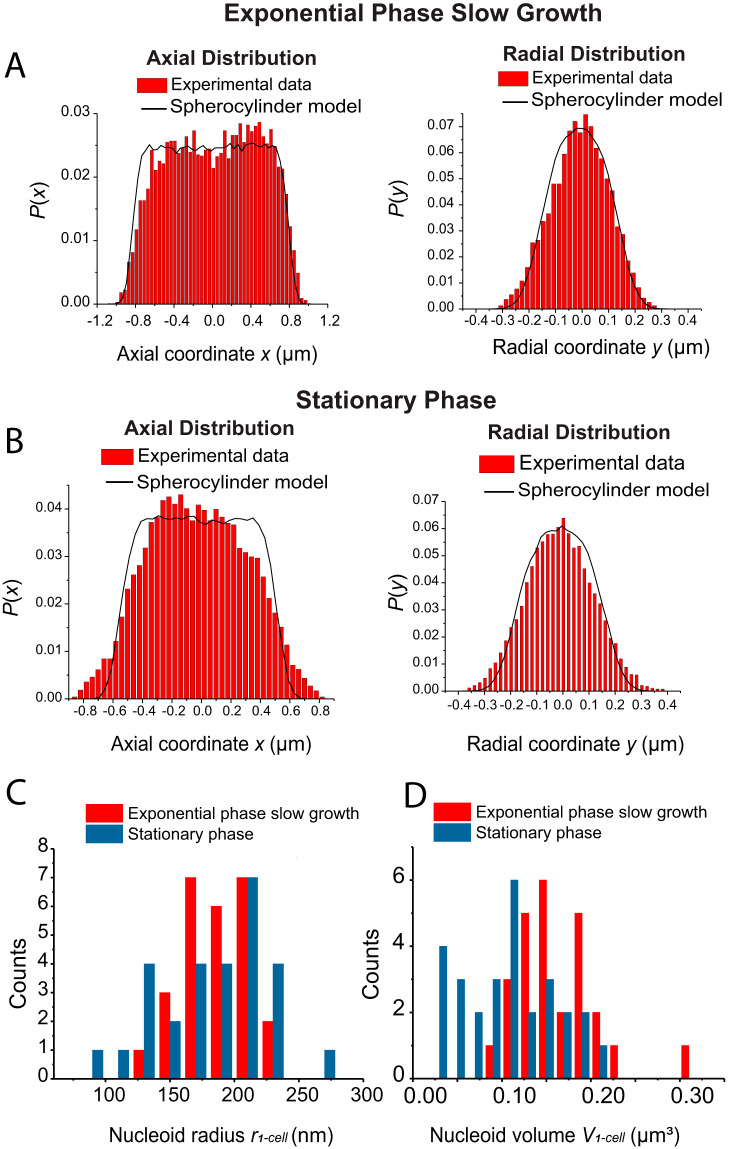
Quantitative estimates of nucleoid radius and volume for cells under slow exponential growth condition (SM6 strain, doubling time 147 min [31]) and in stationary phase. (A) For slow exponential growth, axial (left) and radial (right) distributions of HU (red) were averaged across cells in the length range of 2.3 to 2.5 μm. Radial distributions include only molecules in the nucleoid region ($\left|x\right|$ < 0.5 μm). Black lines represent simulated axial and radial projections of particles uniformly distributed within a spherocylinder of radius *r*_avg_ = 0.18 μm and tip-to-tip length *L *= 1.70 μm. (B) For stationary phase, axial (left) and radial (right) distributions of HU (red) were averaged across cells in the length range of 2.3 to 2.5 μm. Only cells with a single nucleoid lobe are included. Radial distribution includes only molecules in the nucleoid region ($\left|x\right|$ < 0.5 μm). Black lines represent simulated axial and radial projections of particles uniformly distributed within a spherocylinder of radius *r*_avg_ = 0.21 μm and tip-to-tip length of single-lobe nucleoid *L *= 1.20 μm. (C) Distribution of single-cell nucleoid radii for slow exponential growth and for stationary phase. For each cell, the radius was determined by fitting the HU nucleoid axial and radial distributions with calculated projections from a uniform distribution within a spherocylinder. Radius and length were adjusted to find the best fit. (D) Corresponding distributions of single-cell nucleoid volumes.

Next, we estimated the mean volume of a single-lobe nucleoid in 2-day stationary-phase cells lying in the 2.3 to 2.5 μm length range. The averaged HU-PAmCherry spatial distributions of Fig. 2B enable us to calculate a population-weighted average nucleoid volume (31). We fit the averaged radial distribution *P*(*y*) to a simulated distribution for a uniformly filled spherocylinder of radius *r*_avg_ (Fig. 7). The simulation incorporates the estimated location uncertainty σ = 51 nm. The best fit yields *r*_avg_ = 0.21 μm. We used the average tip-to-tip length of a single nucleoid lobe *L*_avg_ = 1.20 μm to estimate the volume of a spherocylinder having these dimensions as *V*_avg_ = 0.15 μm^3^. Dividing *N*_avg_ by *V*_avg_ yields an estimate of the average DNA density in stationary-phase cells as ρ_avg_ ∼4.8 × 10^7^ bp/μm^3^ (Table 2). In the Discussion, we compare this estimate with that derived from an earlier study of exponential-phase cells that are growing very slowly (doubling time 147 min) (31).

**TABLE 2:** Nucleoid parameter estimates from cell-averaged HU distributions^*a*^

Growth condition	*r*_avg_ (μm)	*L*_avg_ (μm)	*V*_avg_ (μm^3^)	ρ_avg_ (bp/um^3^)
Two-day stationary phase	0.21	1.20	0.15	4.8 × 10^7^
Exponential-phase slow growth (147 min)	0.18	1.70	0.16	2.9 × 10^7^

a Stationary-phase data include 28 cells with a single nucleoid lobe in the 2.3-to-2.5-μm length range. Slow-exponential-growth data include 26 cells with a single nucleoid lobe in the 2.3-to-2.5-μm length range. The doubling time was 147 min. Data from reference 31 were reanalyzed here.

However, such a ratio of two averages becomes less representative of the population in the face of substantial cell-to-cell heterogeneity in HU-PAmCherry radial distribution widths. Such heterogeneity is strong in stationary-phase cells, even when restricted to the 2.3 to 2.5 μm length range. Not only do the widths of individual cell distributions vary; in addition, the long axes of individual distributions are not necessarily well centered in the cell body, causing broadening of the averaged *P*(*y*).

As an alternative approach, for the 28 cells in the length range 2.3 to 2.5 μm that exhibit a single nucleoid lobe we can use the same sphereocylinder model to obtain an estimate of each single-cell DNA radius *r*_1−cell_ and each length *L*_1−cell_. This enables us to estimate each single-cell nucleoid volume *V*_1−cell_. The distributions of *r*_1−cell_ and *V*_1−cell_ are shown in Fig. 7C and D. The mean values are <*r*_1−cell_> = 0.18 ± 0.04 μm (± one standard deviation) and <*L*_1−cell_> *=* 1.14 ± 0.22 μm.

The mean single-cell volume is <*V*_1−cell_> = 0.11 ± 0.05 μm^3^ (Table 3), which is 73% of the value *V*_avg_ = 0.15 μm^3^ obtained from the cell-averaged HU distribution. We cannot calculate single-cell DNA densities because we lack a strain enabling both measurement of HU distributions and counting of the *Right2* loci in the same individual cell. Instead, we estimate what a relatively high-density ρ_large_ and what a relatively low-density ρ_small_ could be, consistent with what is known. Dividing two full chromosomes of DNA by the relatively low volume *V*_small_ = 0.11 − 0.05 μm^3^ = 0.06 μm^3^ yields ρ_large_ of ∼1.5 × 10^8^ bp/μm^3^. Dividing one chromosome of DNA by the relatively high volume *V*_large_ = 0.11 + 0.05 μm^3^ = 0.16 μm^3^ yields ρ_small_ of ∼2.9 × 10^7^ bp/μm^3^. Encouragingly, the initial cell-averaged estimate ρ_avg_ of ∼4.8 × 10^7^ bp/μm^3^ lies about halfway between ρ_small_ and ρ_large_.

**TABLE 3:** Nucleoid parameter estimates from averages over single-cell HU distributions^*a*^

Growth condition	Values^*b*^		
	<*r*_1−cell_> (μm)	<*L*> (μm)	<*V*_1−cell_> (μm^3^)
Two-day stationary phase	0.18 ± 0.04	1.14 ± 0.22	0.11 ± 0.05
Exponential-phase slow growth (147 min)	0.18 ± 0.03	1.65 ± 0.11	0.16 ± 0.05

a Stationary-phase data include 28 cells with a single nucleoid lobe in the 2.3-to-2.5-μm length range. Slow-exponential-growth data include 26 cells with a single nucleoid lobe in the 2.3-to-2.5-μm length range. The doubling time was 147 min. Data from reference 31 were reanalyzed here.

b The range of values indicated is ±1 standard deviation (SD).

There is a third approach to this problem. The analysis described above was restricted to stationary-phase cells of length 2.3 to 2.5 μm. The very shortest cells in the distribution of Fig. 1C (length of <2.0 μm) all had one HU lobe (Fig. S3C) and a high probability of only one chromosome (Fig. S3D). Their nucleoid radii and lengths tend to be minimal. Several such cells can be seen in Fig. 1B. For 7 of such cells, we estimated single-cell radii and volumes as described above. For these shortest cells, the mean nucleoid volume is <*V*_1−cell_> = 0.08 ± 0.03 μm^3^. Dividing 1 chromosome = 4.6 × 10^6^ bp by this volume yields the estimate <ρ> = 5.8 × 10^7^ bp/μm^3^, very similar to the result for the mid-range cells.

## DISCUSSION

As summarized in the introduction, the biochemistry of stationary-phase E. coli is reasonably well understood (1, 2, 4). Much less is known about what we would call the biophysical state of the cytoplasm in stationary phase. Here, we have used superresolution fluorescence microscopy to characterize quantitatively the spatial distributions and diffusive properties of the chromosomal DNA, ribosomes, RNAP, and the exogenous Kaede tetramer for E. coli in 2-day stationary phase. Although all the cells are in the same nominal growth stage, the degree of cell-to-cell heterogeneity within this population is striking. Cell lengths differ greatly (Fig. 1C), consistent with previous observations (22). To minimize the effects of length heterogeneity, we restricted the length of stationary-phase cells in our quantitative measurements of spatial distributions to the range 2.3 to 2.5 μm. Even so, cells within this narrow range may exhibit either one or two axial nucleoid lobes (imaged by HU), one or two Kaede lobes, and one or two complete chromosomes (counted as *Right2* puncta).

In exponential phase, it is quite clear that the larger, more slowly diffusing 70S polysomes concentrate in the three ribosome-rich regions. The smaller, more rapidly diffusing 50S subunits have access to the nucleoids (30, 31). For L9 labeling in exponential phase, we are tracking 50S ribosomal subunits, which occur primarily as free 50S subunits (∼1,500 kDa) or as 50S subunits incorporated into translating 70S ribosomes (∼2,500 kDa). The predominant species are 70S polysomes of various lengths and very large mass (47). Because free subunits diffuse more rapidly than 70S polysomes and because the fractional populations *f*_fast_ = 0.20 and *f*_slow_ = 0.80 are consistent with the known fraction of ribosomes undergoing translation (31, 35), the assignment of the fast population (*D*_fast_ = 0.16 μm^2^/s) to free 50S polysomes and of the slow population (*D*_slow_ = 0.025 μm^2^/s) to 70S polysomes is well founded (30).

The situation is less clear in stationary phase. An earlier study using the E. coli laboratory strain W3110 carried out ribosome profiling of total cell extracts separated on a sucrose density gradient, varying the time lag between inoculation and measurement (see Fig. 2 in reference 47). For 2-day and 3-day time lags, the predominant components detected were 100S and 70S, with comparable intensities. 50S and 30S were roughly 10% as intense. In our experiments, the 2-day stationary-phase cytoplasm likely contained L9-mEos2 labels within 100S ribosome dimers (∼5,000 kDa), translating 70S monomers and 70S polysomes, free 50S subunits, 70S species inactivated by binding to RaiA, and potentially partially digested ribosomal fragments containing the L9 subunit (7–9, 48). The two-state model yields fractional populations *f*_fast_ = 0.82 and *f*_slow_ = 0.18, quite similar to the fractions in exponential growth. However, the values of the diffusion coefficients *D*_fast_ = 0.60 μm^2^/s and *D*_slow_ = 0.084 μm^2^/s are much larger than the corresponding values in exponential growth. The single-step displacement distributions (Fig. 5B) cannot distinguish so many species from each other. Indeed, each of the fast and slow populations may well contain a mixture of two or more of the components listed above. The much larger best-fit values of the two diffusion coefficients in stationary phase likely arise at least in part from different speciations of the L9 protein copies being tracked. Changes in the composition and structure of the nucleoid and ribosome-rich regions could also affect diffusion.

Nevertheless, the data show clearly that in stationary phase, the L9-containing species that diffuse most slowly are strongly segregated from the nucleoid, especially in the radial coordinate (Fig. 5D). In addition, the best-fit value of *D*_slow_ has increased substantially, suggesting that the components described by *D*_slow_ diffuse more rapidly in stationary phase. Therefore, we suggest that in stationary phase, the ribosomal components that concentrate outside the nucleoid are 100S particles as well as any remaining 70S polysomes. The latter are presumably shorter in length (and diffuse faster) than the 70S polysomes in exponential growth. Exclusion of 100S particles from the nucleoid is consistent with a somewhat condensed chromosomal DNA polymer. If this picture is essentially correct, then the faster-diffusing, presumably smaller ribosomal species that concentrate in the nucleoid (Fig. 5D) probably include 50S subunits and 30S subunits and, possibly, small ribosomal fragments. This in turn mildly suggests that cotranscriptional translation might be feasible within the stationary-phase nucleoid.

The RNAP spatial distribution in stationary phase is slightly longer and significantly wider than that of the chromosomal DNA (Fig. 2B). Evidently, nonspecific binding of RNAP to DNA remains sufficiently strong and frequent to sequester the RNAP within a volume only moderately larger than that of the nucleoid (and much smaller than the entire cytoplasm). RNAP avoids the ribosome-rich endcaps and the annular region surrounding the DNA. This would enable transcription of the appropriate genes no matter where they are located; there is no need to invoke preferential relocation of active genes to the nucleoid surface. We found no evidence of a hollow RNAP or HU distribution, such as might occur if the DNA had a supercompacted, dense core that was impenetrable to these species.

Surprisingly, the population-averaged RNAP diffusion coefficient is twice as large in stationary phase as in exponential phase (Fig. 4A to C). We suggest two possible underlying causes. First, each RNAP copy may undergo less frequent binding, both specific and nonspecific, to the chromosomal DNA. This is consistent with the length and width of the RNAP distribution being larger than those of DNA. RNAP diffusion may be faster and sequestration weaker due to occlusion of DNA binding sites by the highly abundant Dps protein, whose copy number can be more than 100,000 in stationary-phase cells (10). Second, the abundance of Dps causes formation of micro- or nanocrystals comprising dodecameric Dps oligomers, Mg^2+^, and the chromosomal DNA (12, 13). If this occurred widely throughout the nucleoid, the resulting local bundling of DNA strands could open up a network of interconnected channels within the overall nucleoid volume. Such channels might enable RNAP to penetrate the interior of the nucleoid readily, diffuse relatively rapidly there, and locate and transcribe important genes. Both of these mechanisms could operate at the same time.

The local jiggling motion of the DNA locus *Right2* is attenuated 4-fold in stationary phase compared with exponential phase, as judged by the apparent diffusion coefficient *D*_app_ on the 10-s timescale (Fig. 3B). It seems sensible that DNA coated with Dps and partially organized into micro- or nanocrystals would be stiffer (more rigid) than in exponential growth, where most of the DNA is free of binding proteins (12, 13). In addition, the lower overall level of metabolic activity in stationary phase attenuates the amount of ATP-driven activity by motor proteins. In exponential growth, such activity evidently drives much of the subdiffusive motion of DNA loci (29, 49). Nevertheless, *D*_app_ remains at least 7 times larger in stationary phase than in cells harvested from cultures in exponential growth and then chemically fixed (34).

Meanwhile, the nucleoid of a 2-day stationary-phase cell has, on average, volume comparable to and DNA density only about 2-fold higher than the levels estimated by analysis of data from an earlier study of very slowly growing exponential-phase cells (doubling time of 147 min at 30°C; Table 2) (31). This comparison is appropriate because in both cases the DNA spatial distribution appears smooth on an ∼100-nm length scale and is reasonably well modeled as a uniformly filled spherocylinder. In stationary phase, RNAP (450 kDa), Kaede tetramer (110 kDa), and the faster-moving L9-containing ribosomal species (likely including 50S subunits of 1,500-kDa mass) all penetrate the nucleoid and indeed concentrate there. The 2-day stationary-phase nucleoid might best be described as “moderately compacted” compared with the nucleoids in slow exponential growth.

## MATERIALS AND METHODS

### Strains, cell growth, and preparation for imaging.

The strains used in the study are listed in Table 1. The SB1 strain (ribosome L9-mEos2) was a gift from the Sanyal lab at Uppsala University (22). The construction details of each strain are described in the corresponding references.

For imaging of cells in both the exponential and stationary phases, 2 ml of bulk culture was first grown from glycerol frozen stock to overnight stationary phase in EZ rich defined medium (EZRDM) at 30°C. EZRDM is a morpholinepropanesulfonic acid (MOPS)-buffered solution at pH = 7.4 supplemented with metal ions (M2130; Teknova), glucose (2 mg/ml), amino acids and vitamins (M2104; Teknova), nitrogenous bases (M2103; Teknova), 1.32 mM K_2_HPO_4_, and 76 mM NaCl. For imaging of exponential-phase cells, subcultures were grown to an optical density (OD) at 600 nm of 0.2 to 0.6 at 30°C before sampling for the microscopy experiments. For imaging of 2-day stationary-phase cells, several replicates of the same strain were grown at the same time. For each replicate, 10 μl of overnight culture was diluted into 2 ml of fresh EZRDM. After 48 h, one group of replicates was used for imaging. The other group was centrifuged and the supernatant collected, filtered, and warmed to 30°C, providing what we refer to here as “spent medium.” The spent medium was flowed across plated stationary-phase cells to maintain the stationary phase during imaging. The analysis of the nucleoid volume for cells in slow exponential growth (147 min doubling time) was carried out on raw data from reference 31.

Strains that express labeled species from a plasmid (Table 1) were grown with the addition of 100 μg/ml ampicillin. For exponential-phase imaging, when the cells reached mid-log phase, anhydrotetracycline was added to reach a final concentration of 45 nM to induce the expression of the labeled protein of interest. After 10 min of induction, the cells were centrifuged and resuspended in fresh growth media with 100 μg/ml ampicillin to remove the inducer. The cells were then incubated again in growth media for 15 min at 30°C to enable maturation of the labeled protein of interest prior to imaging. For stationary-phase imaging, 100 μl of 48-h stationary-phase culture was added to 900 μl of spent medium. Anhydrotetracycline was added to the culture to reach a final concentration of 45 nM. After 10 min of induction, the cells were centrifuged twice and resuspended in spent medium with 100 μg/ml ampicillin to remove the inducer. The cells were then incubated again in spent medium for 15 min at 30°C.

For nucleoid staining experiments in exponential-phase cells, Sytox Green dye was added to a growing mid-log-phase culture (OD at 600 nm = 0.2 to 0.6) to reach a final concentration of 500 nM (50). After 10 min of incubation, the cells were centrifuged twice and resuspended in fresh EZRDM before imaging. The rinsing steps eliminated background fluorescence from dye molecules that adhered to the coverslip without removing Sytox Green from the cytoplasm. For nucleoid staining experiments in stationary-phase cells, 100 μl 48-h stationary-phase culture was added to 900 μl of spent medium. Then, Sytox Green was added to reach a final concentration of 10 μM. After 30 min of incubation, the cells were centrifuged twice and resuspended in spent medium before imaging. Penetration of the membranes of stationary-phase cells by Sytox Green is relatively inefficient. To provide good images, the concentration of Sytox Green was higher and the incubation time was longer.

For both growth phases, ∼150 μl of cell subculture was placed within a CoverWell perfusion chamber gasket (Invitrogen) on a polylysine-coated, cleaned coverslip to fill the entire chamber volume. We allowed 2 min for the cells to adhere to the coverslip. The plated cells were then rinsed with the appropriate fresh, warmed, aerated media to wash away any nonadhered cells. For cells in exponential growth, the rinsing medium used was EZRDM. Cells continued to grow normally for at least 30 min under these conditions. For stationary-phase cells, the rinsing medium was spent medium. Cells were maintained at 30°C throughout the imaging using an automatic temperature controller.

Stationary-phase cells plated in a microfluidic device with a flow of spent medium do not grow for at least 1 h. On switching the flow to fresh EZRDM, cells of all lengths begin to grow within ∼1 min (see Fig. S1 in the supplemental material). In a different experiment, we added 10 μl of stationary-phase cell culture to 2 ml of spent medium. As measured by OD, the cells did not grow for at least 24 h.

For studying exponential-phase cells under ATP-depleting conditions, cells were treated with 200 μM carbonyl cyanide-*m*-chlorophenylhydrazone (CCCP) plus 1 mM 2-deoxyglucose (37, 40, 49, 51) for 10 min, prior to imaging. During the imaging, EZRDM was supplemented with each drug at the same concentration (34).

### Microscopy.

Reasonably high-quality heat maps and histograms require localization of hundreds of single copies. In some cases, it would be possible to obtain a large number of localizations from a single cell, but we have found that the laser dosages required to do so typically kill E. coli. Instead, we prefer to limit our quantitative studies to a narrow length range and combine data from many cells that have each experienced a much lower laser dosage.

All imaging was performed on a Nikon Eclipse Ti inverted microscope (Nikon) with an oil immersion 100×, 1.45 numerical-aperture (N.A.) phase-contrast lens objective (CFI Plan Apo Lambda DM; Nikon Instrument). The images were further magnified 1.5×. Fast shutters (Uniblitz LS2; Vincent Associates) were used to synchronize illumination and image acquisition. Except for Kaede, images were recorded by the use of a back-illuminated electron-multiplying charge-coupled device (EMCCD) camera with 16-μm-by-16-μm pixels (either an Andor iXon DV-897 or an Andor iXon DV-887 camera; Andor Technology). Each pixel corresponds to 105 nm × 105 nm at the sample with an overall magnification of ×150. Kaede images were recorded by the use of an Andor iXon 860 camera, with each pixel corresponding to 160 nm × 160 nm.

For superresolution imaging of RNAP β′-mEos2 (strain HC1), HU-PAmCherry (strain SM7), ribosomes labeled by L9-mEos2 (strain SB1), ribosomes labeled by S2-mEos2 (strain MSG196), and Kaede (strain JCW 96), the fluorescent protein was photoconverted using a 405-nm laser at ∼4 to 12 W/cm^2^ and subsequently imaged using a 561-nm-wavelength excitation laser at ∼2 kW/cm^2^. The emission filter used was model ET610/75 (Chroma Technology). RNAP β′-mEos2, HU-PAmCherry, ribosome L9-mEos2, and ribosome S2-mEos2 were imaged at a frame rate of 31.2 Hz, with an exposure time of 30 ms. Kaede was imaged with a frame rate of 485.4 Hz, with an exposure time of 2 ms.

Images of the DNA stain Sytox Green were collected at 30 ms/frame with 514-nm laser power density of ~10 W/cm^2^. Dichroic ZT405-514-561rpc (Chroma) and filter HQ525/50 (Chroma Technology) were used.

Studies of the motion of the *Right2* DNA loci labeled by ParB-GFP (strain JCW154) used 488-nm excitation (Coherent Sapphire laser, ∼100 W/cm^2^ at the sample), with the laser beam expanded to illuminate the field of view uniformly. The emission filter used was model HQ525/50 (Chroma Technology). The labeled protein ParB-GFP polymerizes specifically at a *parS* site engineered into the chromosome near the *Right2* locus, forming bright puncta. The loci could be tracked with a good signal-to-noise ratio for 600 camera frames at 1 s/frame and 50-ms exposure time.

### Image analysis for spatial distribution.

Images were analyzed using a MATLAB graphical user interface (GUI) developed in our lab (45). Images were smoothed and filtered to obtain a zero-based digital image. Bright spots were located with pixel-level accuracy by a peak finding algorithm that detects the local intensity maxima within an image. A user-defined intensity threshold was used as the minimum brightness of a pixel arising from a single molecule. The threshold is carefully set by the user so that it will not be so high as to reject a real single molecule in the raw images or so low as to include background noise.

To obtain the tip-to-tip cell length and to define the (*x*, *y*) coordinates of each particle within the cell, cell outlines were generated from phase-contrast images by the use of Oufti open-source image analysis software (32). The Oufti mesh generally overestimates cell length (Fig. S2B). To generate the spatial distribution of molecules, the camera-based coordinates were reoriented so that the *x* axis and *y* axis corresponded to the long and short principal cell axes.

Additional details of the analysis of diffusive behavior are provided in the supplemental material.
