# Supplementary figures and images for: Biophysical Properties of Escherichia coli Cytoplasm in Stationary Phase by Superresolution Fluorescence Microscopy
Source: mBio. 2020 Jun 16;11(3):e00143-20. doi: 10.1128/mBio.00143-20 (PMC7298701; doi:10.1128/mBio.00143-20)

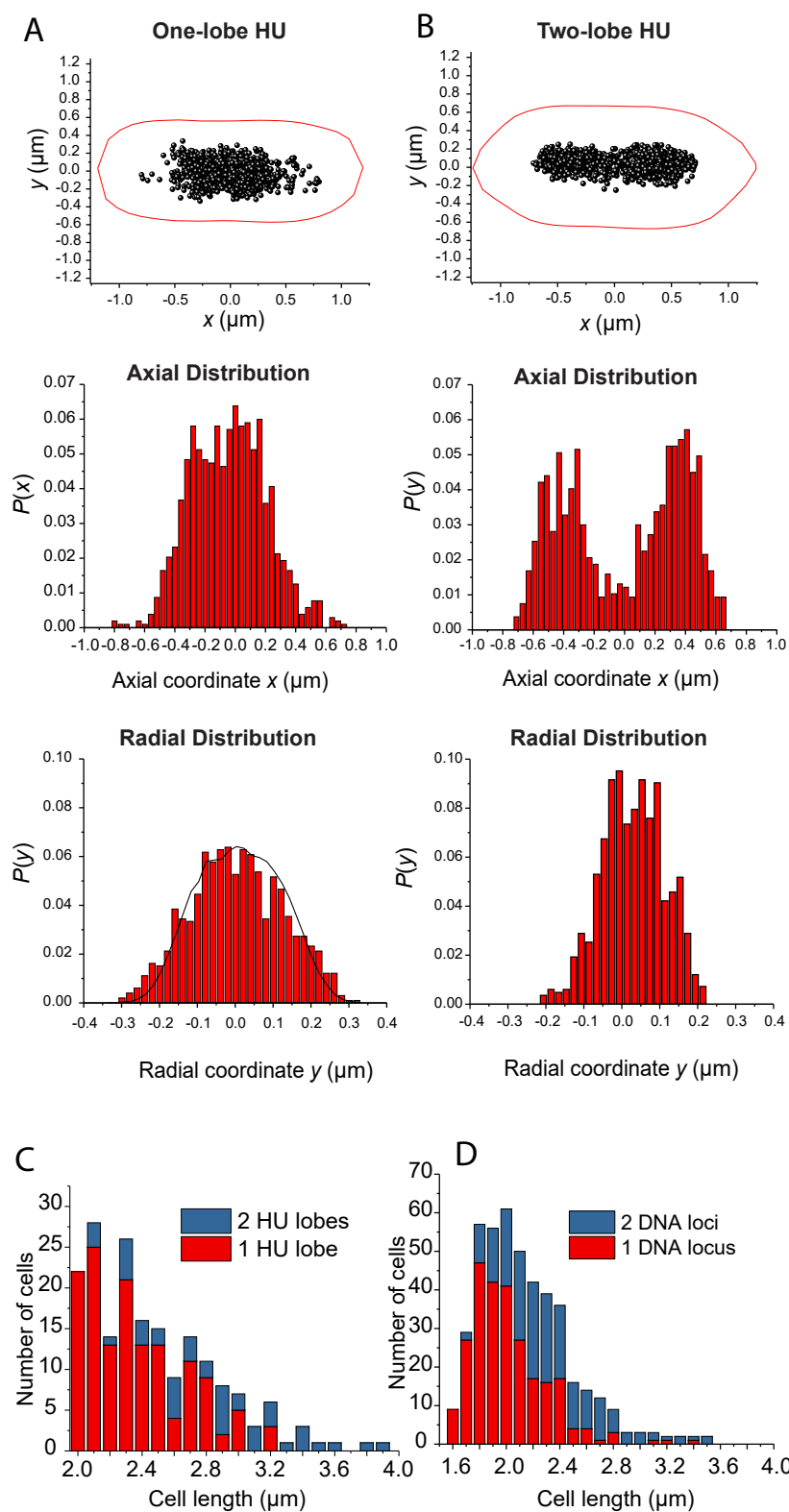

Supplement: FIG S3 [file mBio.00143-20-sf003.pdf]
