# Supplementary material for: Biophysical Properties of Escherichia coli Cytoplasm in Stationary Phase by Superresolution Fluorescence Microscopy
Source: mBio. 2020 Jun 16;11(3):e00143-20. doi: 10.1128/mBio.00143-20 (PMC7298701; doi:10.1128/mBio.00143-20)
Supplement: TEXT S1 [file mBio.00143-20-s0001.docx]

**Supporting Materials**

**Image analysis for tracking**

A modified MATLAB version of the tracking program written by Crocker and Grier (1) was used. As before (2), a centroid algorithm was used to locate the identified particles with subpixel resolution. Centroids of the bright spots were calculated from a 7 x 7 pixel square containing the entire bright spot, centered on the local maximum determined by the peak finding algorithm. The centroid positions from successive frames were connected to form a trajectory. For ribosomal, RNAP and HU trajectories, the centroid positions are connected only if they lie within
4 px = 420 nm of each other. For Kaede, the centroid positions are connected only if they lie within 5 px = 800 nm. For ribosomes, RNAP and HU, each pixel is 105 nm x 105 nm, while for Kaede each pixel is 160 nm x 160 nm.

In each experimental condition, the ensemble-averaged mean-square displacement was calculated as:

$MSD\left( \tau\right)=\left\langle\left( \vec{R}\left( t+\tau\right)-\vec{R}\left( t \right) \right)^{2} \right\rangle=\frac{1}{N}\sum_{i} \frac{1}{m}\sum_{m} {[\vec{R}_{i}\left( mt+\tau\right)-\vec{R}_{i}\left( mt \right)]}^{2}.$ (S1)

Here *N* is the number of trajectories over which the ensemble average is taken. The index *m* runs from 1 to a specific value given the lag time $\tau$, providing the time average of each trajectory. The final MSD is an average over the ensemble and over time. For DNA loci imaged with 1 s/frame time, *m* runs from 1 to 99. We used the linear fit of the first 10 points on the MSD plots to calculate an approximate apparent diffusion coefficient*.* For other particles including RNAP β’–mEos2, ribosomal S2-mEos2, ribosomal L9-mEos2, HU-PAmCherry, and Kaede, we chose trajectories that lasted at least 6 steps (7 frames) to analyze and trajectories longer than
7 frames were truncated to 7 frames. Here *m* runs from 1 to 6 and we used the linear fit of the first 3 points on the MSD plots to calculate an approximate apparent diffusion coefficient via an average over thousands of trajectories (3).

The MSD points at larger time lags are less averaged because there are fewer number of displacements and will thus have larger statistical fluctuations. On the other hand, the uncertainty on the MSD points at small time lag could be high in the presence of localization error. Michalet has analytically addressed this MSD analysis with localization error and discussed the optimal number of points for fitting (3). He introduced the reduced localization error *x* $=\frac{\sigma2}{D\Delta t}$ ,
where *σ, D,* Δ*t* are localization error, diffusion coefficient and frame time respectively and proposed that the best estimate of the diffusion coefficient is obtained from the first two points when $x$ << 1 while a larger number of MSD points are needed for fitting when $x$ >>1.The initial slope of MSD plots for the DNA loci should be interpreted differently from those for ribosomes and HU. The DNA loci motion is a composite of “jiggling in place” (sub-diffusive) and longer-term movement of the average location. For DNA loci, the MSD plot has a relatively small positive slope, especially for fixed cells. Linear fitting of the first 3 points incorporates very large localization error into the estimate of *D_app_*. Somewhat arbitrarily, for DNA loci we fit the first 10 data points to a straight line to estimate *D_app_*.

For ribosomes, HU, and Kaede, the motion may involve confinement, causing curvature of the MSD plot at longer lag times. We choose to fit the first three data points because the noise increases as the lag time increases. Suppose the best least-squares, linear fit to the first experimental points is given by the equation MSD(τ) = *c* + *kτ*, with *k* the slope and *c* the extrapolated intercept at lag time *τ* = 0. Then Michalet (3) has shown that the most accurate mean diffusion coefficient is given by *D* = *k*/4 and the best estimate of the dynamic localization error is σ = ½ (c + 4*Dt*_E_/3)^1/2^, where *t*_E_ is the exposure time within each camera frame.

**Statistical test for significant differences in MSD slopes**

To test for significant differences between two values of *D_app_* obtained under different experimental conditions, we applied a two-tailed Student’s *t*-test of the null hypothesis *H*_0_ that the statistic *T* = (*k­­_A_* – *k_B_*) = 0. Here *k_A_* and *k_B_* are the two least-squares fits to the MSD(τ) slopes for data sets *A* and *B*. Details are provided in Ref. (4). Consider two sets of MSD data (*x_Ai_*,*y_Ai_*), (*x_Bi_*,*y_Bi_*) with best-fit slopes *k_A_* and *k_B_*. Here *x* corresponds to the lag time *τ*, *y* corresponds to the MSD, and *i* runs from 1 to *n*, with *n* the number of points used for fitting. Thus *n_A_* = *n_B_* = 3 for MSD plots for RNAP, ribosomes, HU and Kaede; and *n_A_* = *n_B_* = 10 for MSD plots for DNA loci. The experimental *t*-value is then

*t_expt_* = $\frac{(k_{A}-k_{B})}{\sqrt{{{SE}_{k_{A}}}^{2}+{{SE}_{k_{B}}}^{2}}}$ , where

$${SE}_{k_{A}}=\frac{\sqrt{\frac{\sum{(y_{Ai}-\bar{y_{A}})}^{2}-\frac{[\sum{(x_{Ai} - \bar{x_{A}})(y_{Ai} - \bar{y_{A}})]}^{2}}{\sum{(x_{Ai}-\bar{x_{A}})}^{2}}}{(n_{A}-2)}}}{\sqrt{\sum{(x_{Ai}-\bar{x_{A}})}^{2}}}$$

and

${SE}_{k_{B}}=\frac{\sqrt{\frac{\sum{(y_{Bi}-\bar{y_{B}})}^{2}-\frac{[\sum{(x_{Bi} - \bar{x_{B}})(y_{Bi} - \bar{y_{B}})]}^{2}}{\sum{(x_{Bi}-\bar{x_{B}})}^{2}}}{(n_{B}-2)}}}{\sqrt{\sum{(x_{Bi}-\bar{x_{B}})}^{2}}}$*.*

The *p* value is obtained from *t­_expt_* and the number of degrees of freedom *df* = *n_A_* + *n_B_* – 4. The *p* value is the probability that the null hypothesis *H*_0_ is true and yet random noise resulted in a value that is *t_expt_* or larger. The smaller the value of *p*, the less likely that *H*_0_ is true, i.e., that
*k_A_* = *k_B_*. In common practice, a significance level α = 0.05 or 0.01 is chosen as the level of statistical significance. In this work we use α = 0.01. The two slopes are said to be statistically different if *p* < *α* (denoted ** in the figures). If *p* ≥ α, then the difference in the two slopes is said not to be statistically significant (denoted “n.s.” in the figures). EXCEL provides the functions STEYX, VAR.S, T.DIST.2T for convenient calculation of *t_expt_* and *p*.

**Monte Carlo simulations to fit experimental *P*(*r*) distributions**

In both exponential phase and stationary phase, experimental trajectories of ribosomes and RNAP that lasted 6 steps or longer were selected for analysis. Longer trajectories were truncated to 6 steps. The 6-step trajectories were then sliced into individual steps. The single-step displacements $r_{i}=\sqrt{({x_{i+1}-x_{i})}^{2}+({y_{i+1}-y_{i})}^{2}}$were pooled to form the experimental distributions *P*(*r*) in Figs. 4 and 5. Many previous studies of single-molecule diffusion fit *P*(*r*) to a sum of analytical functions, each describing one diffusive state (one diffusion coefficient *D*). However, these analytical functions describe free diffusion in an infinite space (2, 5-7). Molecules diffusing rapidly in the *E. coli* cytoplasm suffer from confinement due to the spherocylindrical cell boundaries, a problem for which there is no analytical solution. Instead, we use Monte Carlo simulation to generate a model numerical function *P_model_*(*r*;*D*) describing the behavior of each diffusive state, taking account of confinement effects and the dynamic localization error σ. Each simulated model function describes one diffusive state, with values of *D* and *σ* fixed.

For each *D*, we calculate a large number of random walk trajectories within a model spherocylinder that mimics the dimensions of a typical *E. coli* cell in the corresponding growth condition (8). At *t* = 0, 100,000 particles are randomly distributed within the cell volume. Each particle undergoes a random walk independent of other particle positions. To model each 30-ms camera image, three-dimensional microtrajectories (1000 steps of 30 μs each) were generated. At each time step, each particle chooses a displacement in each of three Cartesian directions. These displacements are chosen from a Gaussian distribution whose standard deviation corresponds to the state’s three-dimensional diffusion coefficient *D*. In the rare event that a particle attempts to step outside of the cell boundaries, the displacement for that microstep is taken to be zero. The location of each particle during each camera frame is obtained as the centroid of the model microtrajectories in order to mimic the analysis procedure used for the experimental images. The appropriate dynamic localization error σ is then applied to each centroid location in both *x* and *y* coordinates by sampling a Gaussian distribution with standard deviation σ. By adding the error to the centroid position we obtain the model “measured” location for each 30 ms camera frame. The *x* and *y* coordinates of each measured location are stored for further analysis. For the next model camera frame, each particle continues to make microsteps in 3D starting from the endpoint of the previous camera frame. By connecting the sequence of measured simulation locations over seven frames, we form 100,000 model trajectories for each relevant value of *D*. These trajectories are used to compute model-based, one-step probability distributions *P_model_*(*r*;*D*) that become the numerical input functions for the least-squares analysis of the corresponding experimental distribution.

We judge the goodness of fit by evaluating the reduced chi-square statistic (9):$\chi_{\nu}^{2}=\frac{1}{\left(N-\alpha\right)}\sum_{j=1}^{N} \frac{(h{}_{j}-y{}_{j})^{2}}{s_{j}^{2}}$. Here *j* labels the *N* bins in the (unnormalized) *P_exp_*(*r*) and *P_model_*(*r*) histograms, *h_j_* is the number of experimental counts in bin *j*, *y_j_* is the number of counts in bin *j* of the simulated *P_model_*(*r*) histogram, $s_{j}^{2}$ is the variance of the value in bin *j*, and α is the number of fitted parameters (10). We take $s_{j}^{2}$ = *h_j_* as the estimate of the variance, assuming Poisson statistics. A good fit to an adequately model function should have χ_ν_^2^ ~ 1.

It was shown previously for cells in exponential growth that according to the reduced chi-square statistic χ_ν_^2^, the ribosome distribution *P*(*r*) is poorly fit by a one-state model (10), *i.e.*, to a single numerical function *P_model_*(*r*;*D*). So we modeled *P*(*r*) as a sum of two static
(non-exchanging) populations: *P_model_*(*r*) = *f_slow_P*(*r*;*D_slow_*) + (1 – *f_slow_*)*P*(*r*;*D_fast_*). It includes a fast diffusing component and a slow diffusing component. The appropriate values of σ*_fast_* and σ*_slow_*, which were estimated from the intercept of MSD(τ) plot, were used for each component (11). The least-squares fitting procedure involved a numerical search for the lowest value of χ_ν_^2^ on a 3D grid of combinations of the three independent adjustable parameters (*D_fast_*, *D_slow_*, *f_slow_*). The fraction of slow component *f_slow_* varies from 0 to 1 in intervals of 0.01.

**References for Supporting Materials**

1. Crocker JC, Grier DG. 1996. Methods of Digital Video Microscopy for Colloidal Studies. Journal of Colloid and Interface Science 179:298-310.

2. Bakshi S, Siryaporn A, Goulian M, Weisshaar JC. 2012. Superresolution imaging of ribosomes and RNA polymerase in live *Escherichia coli* cells. Mol Microbiol 85:21-38.

3. Michalet X. 2010. Mean square displacement analysis of single-particle trajectories with localization error: Brownian motion in an isotropic medium. Phys Rev E Stat Nonlin Soft Matter Phys 82:041914.

4. Cohen J, Cohen J. 2003. Applied multiple regression/correlation analysis for the behavioral sciences. L. Erlbaum Associates,, Mahwah, N.J.

5. Bakshi S, Dalrymple RM, Li W, Choi H, Weisshaar JC. 2013. Partitioning of RNA polymerase activity in live *Escherichia coli* from analysis of single-molecule diffusive trajectories. Biophysical Journal 105:2676-2686.

6. Stracy M, Jaciuk M, Uphoff S, Kapanidis AN, Nowotny M, Sherratt DJ, Zawadzki P. 2016. Single-molecule imaging of UvrA and UvrB recruitment to DNA lesions in living *Escherichia coli*. Nature Communications 7:12568.

7. Chen T-Y, Santiago AG, Jung W, Krzemiński Ł, Yang F, Martell DJ, Helmann JD, Chen P. 2015. Concentration- and chromosome-organization-dependent regulator unbinding from DNA for transcription regulation in living cells. Nature Communications 6:7445.

8. Mohapatra S, Weisshaar JC. 2018. Functional mapping of the E. coli translational machinery using single-molecule tracking. Mol Microbiol doi:10.1111/mmi.14103.

9. Press WH. 2007. Numerical recipes : the art of scientific computing. Cambridge University Press,, Cambridge, UK ; New York.

10. Mohapatra S, Choi H, Ge X, Sanyal S, Weisshaar JC. 2017. Spatial Distribution and Ribosome-Binding Dynamics of EF-P in Live *Escherichia coli*. MBio 8.

11. Zhu YY, Mohapatra S, Weisshaar JC. 2019. Rigidification of the *Escherichia coli* cytoplasm by the human antimicrobial peptide LL-37 revealed by superresolution fluorescence microscopy. P Natl Acad Sci USA 116:1017-1026.

**Figure Legends for Supporting Materials**

**Figure S1.** Recovery of three single stationary phase cells after restoration of growth medium. Stationary phase cells were plated in the microfluidic device. Spent medium flowed for the first 30 min. At t = 0, the flow was switched to fresh, aerated EZRDM. Phase contrast images were acquired every 12 s to measure cell length. L/L_0_ is the cell length vs time, normalized to its length at t = –30 min (i.e., long before the flow was changed). There is no evidence of growth while flowing spent medium.

**Figure S2. (A)** Cell length distributions from phase contrast images of different strains in two-day stationary phase. **(B)** Correlation of cell widths as determined by two different methods: the Oufti mesh drawn for phase contrast images and the distance between the two peaks of a transverse line scan through a fluorescence image of the cell outline from the membrane binding dye FM4-64. The vertical lines mark the mean width of single-cell Kaede distributions, taken to be twice the best-fit radius to the cylindrical model. The resulting Kaede widths are 0.82 ± 0.04 μm in exponential phase and 0.50 ± 0.12 μm in stationary phase. It is plausible that Kaede fills the cytoplasm in exponential phase, but its distribution is much narrower than the cytoplasm in stationary phase. **(C)** Distributions of aspect ratios (length/width from Oufti cell outlines derived from phase contrast images) in stationary phase and exponential growth (47 min doubling time).

**Figure S3. (A, B)** Examples of single-cell DNA (HU-PAmCherry) spatial distributions exhibiting one axial lobe (A) or two axial lobes (B). Top: scatter plot of HU locations. Red line is cell mesh generated from phase contrast image using Oufti program. Middle: axial distribution of HU locations. Bottom: radial distribution of HU locations. Each radial distribution includes only molecules in the nucleoid region ($\left| x \right|$< 0.5 μm for one-lobed cell and 0.2 μm < $\left| x \right|$< 0.6 μm for two-lobed cell). Black line: simulated radial projection of particles uniformly distributed within a spherocylinder of radius r_1-cell_ = 0.20 μm. **(C)** Number of cells with one or two axial HU lobes as a function of cell length. **(D)** Number of cells with one or two DNA loci by counting Right2 puncta as a function of cell length.

**Figure S4.** Cell-averaged HU-PAmCherry MSD vs lag time plots from movies taken at 30 ms/frame for exponential phase and stationary phase cells. Apparent diffusion coefficient D_app_ from linear fitting of first three data points. The numerical results are D_app_ = 0.098 ± 0.004 μm^2^/s in exponential phase and D_app_ = 0.088 ± 0.004 μm^2^/s for stationary phase. Localization error σ is 54 nm in exponential phase and 51 nm in stationary phase. For a linear least-squares fit of the data up to τ = 6 steps, R^2^ = 0.998 for exponential phase and R^2^ = 0.997 for stationary phase. Statistical testing (see above) found that the two values of D_app_ are not statistically different
(p = 0.26).

**Figure S5.** Spatial distribution and mean diffusion of ribosomal species labeled with the S2-mEos2 and L9-mEos2 constructs. **(A)** 2D location heat map averaged across cells in the length range 2.3–2.5 μm in stationary phase. Pixel size is 40 nm × 40 nm. Color scale in probability per pixel, normalized so that sum is 1. Scale bar: 1μm. **(B)** MSD vs lag time τ from trajectories taken at 30 ms per frame in four different cases: L9 and S2 labeling in both exponential growth and two-day stationary phase. The apparent diffusion coefficient *D_app_* is obtained by linear fitting of the first three data points. The numerical results are: *D_app_* = 0.035 ± 0.001 μm^2^/s for L9 labeling in exponential growth; *D_app_* = 0.080 ± 0.002 μm^2^/s for L9 labeling in stationary phase; *D_app_* = 0.042 ± 0.001 μm^2^/s for S2 labeling in exponential growth; and *D_app_* = 0.151 ± 0.002 μm^2^/s for S2 labeling in stationary phase. Estimated localization errors are 32 nm, 41 nm, 34 nm, and 47 nm respectively. For a linear least-squares fit of the data up to τ = 6 steps, *R*^2^ values are 0.997, 0.998, 0.999, and 0.998 respectively. Exponential phase data of S2 labeling from Ref (11). In stationary phase, *D_app_* for cells labeled by S2-mEos2 is almost twice that of cells labeled by L9-mEos2. Statistical testing (see above) found no significant difference between S2 and L9 labeling in exponential phase (*p* = 0.21). Difference between S2 and L9 labeling in stationary phase was statistically significant (*p* = 0.007). Difference between S2 labeling in stationary vs exponential phases was also statistically significant (*p* = 0.002). **(C)** Axial (*left*) and radial (*right*) distribution of ribosome in stationary phase cells with length of 2.3–2.5 μm. Radial distribution includes only molecules in the nucleoid region ($\left| x \right|$< 0.5 μm).

**Figure S6.** One-lobe and two-lobe Kaede spatial distributions in stationary phase cells.
**(A)** Example of a one-lobe Kaede cell. *Left*: Scatter plot of Kaede locations. Red line is cell mesh generated from phase contrast image using Oufti. *Middle* *and Right*: axial and radial projections of Kaede distribution for this cell. Radial distribution includes only molecules in the nucleoid region ($\left| x \right|$< 0.5 μm). *Black line*: simulated radial projections of particles uniformly distributed within a spherocylinder of radius *r_1-cell_* = 0.27 μm. **(B)** Example of a two-lobe Kaede cell. Scatter plot, axial distribution, and radial distribution as shown. Radial distribution includes only molecules in the nucleoid region ($\left| x \right|$< 0.5 μm). **(C)** Number of cells with one or two Kaede axial peaks as a function of cell length. **(D)** Cell-averaged axial distributions for stationary phase cells having one or two lobes of Kaede and for stationary phase cells having one or two axial lobes of DNA (HU-PAmCherry), each plotted separately for comparison. **(E)** Kaede axial distribution averaged over cells in stationary phase for the specific length ranges shown. Axial distribution averaged over exponentially growing cells included for comparison.
