# Supplementary material for: Biophysical Properties of Escherichia coli Cytoplasm in Stationary Phase by Superresolution Fluorescence Microscopy
Source: mBio. 2020 Jun 16;11(3):e00143-20. doi: 10.1128/mBio.00143-20 (PMC7298701; doi:10.1128/mBio.00143-20)
Supplement: FIG S6 [file mBio.00143-20-sf006.pdf]

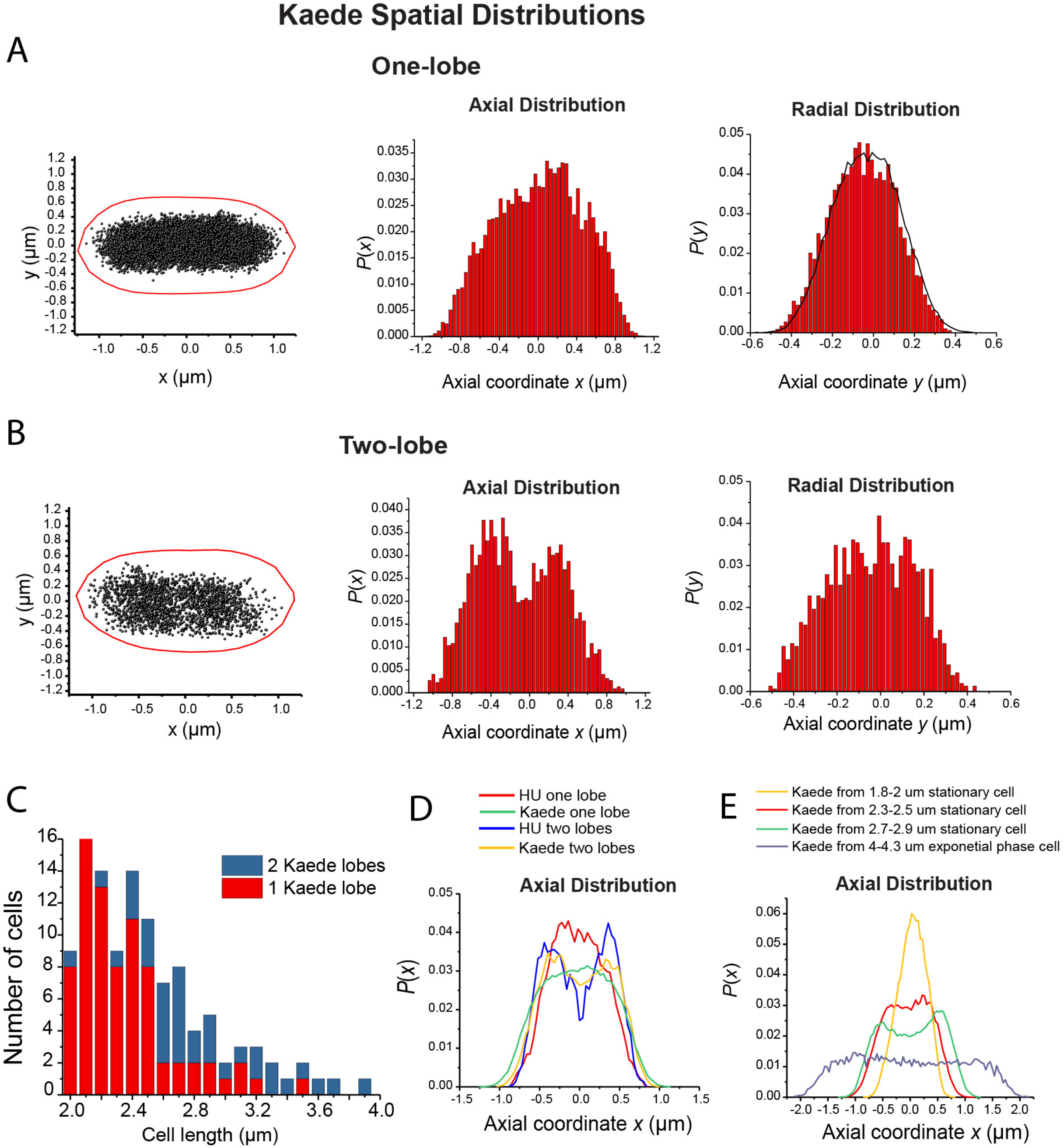
Kaede Spatial Distributions
A
One-lobe
Axial Distribution
Radial Distribution
y (µm)
x (µm)
P(x)
Axial coordinate x (µm)
P(y)
Axial coordinate y (µm)
B
Two-lobe
Axial Distribution
Radial Distribution
y (µm)
x (µm)
P(x)
Axial coordinate x (µm)
P(y)
Axial coordinate y (µm)
C
2 Kaede lobes
1 Kaede lobe
Number of cells
Cell length (µm)
D
HU one lobe
Kaede one lobe
HU two lobes
Kaede two lobes
Axial Distribution
P(x)
Axial coordinate x (µm)
E
Kaede from 1.8-2 um stationary cell
Kaede from 2.3-2.5 um stationary cell
Kaede from 2.7-2.9 um stationary cell
Kaede from 4-4.3 um exponetial phase cell
Axial Distribution
P(x)
Axial coordinate x (µm)
